# Preparedness for colorectal cancer surgery and recovery through a person-centred information and communication intervention – A quasi-experimental longitudinal design

**DOI:** 10.1371/journal.pone.0225816

**Published:** 2019-12-12

**Authors:** Joakim Öhlén, Richard Sawatzky, Monica Pettersson, Elisabeth Kenne Sarenmalm, Cecilia Larsdotter, Frida Smith, Catarina Wallengren, Febe Friberg, Karl Kodeda, Eva Carlsson

**Affiliations:** 1 Institute of Health and Care Sciences and University of Gothenburg Centre for Person-Centred Care, Sahlgrenska Academy at the University of Gothenburg, Gothenburg, Sweden; 2 Palliative Centre, Sahlgrenska University Hospital Västra Götaland Region, Gothenburg, Sweden; 3 School of Nursing, Trinity Western University, Langley, BC, Canada; 4 Centre for Health Evaluation and Outcome Sciences, Vancouver, BC, Canada; 5 Vascular Department, Sahlgrenska University Hospital, Gothenburg, Sweden; 6 Research & Development Unit, Skaraborg Hospital, Skövde, Sweden; 7 Department of Nursing science, Sophiahemmet University, Stockholm, Sweden; 8 Center for Health Care Improvement, Department of Technology Management and Economics, Division of Service Management and Logistics, Chalmers University of Technology, Gothenburg, Sweden; 9 Regional Cancer Center West, Sahlgrenska University Hospital, Gothenburg, Sweden; 10 Faculty of Health Sciences, University of Stavanger, Stavanger, Norway; 11 Department of Surgery, Institute of Clinical Sciences, Sahlgrenska Academy at the University of Gothenburg, Gothenburg, Sweden; 12 Department of Surgery, Sahlgrenska University Hospital/Östra, Gothenburg, Sweden; University of Washington, UNITED STATES

## Abstract

To meet patients’ information and communication needs over time in order to improve their recovery is particularly challenging for patients undergoing cancer surgery. The aim of the study was to evaluate whether an intervention with a person-centred approach to information and communication for patients diagnosed with colorectal cancer undergoing surgery can improve the patients’ preparedness for surgery, discharge and recovery during six months following diagnosis and initial treatment. The intervention components involving a novel written interactive patient education material and person-centred communication was based on critical analysis of conventional information and communication for these patients. During 2014–2016, 488 consecutive patients undergoing elective surgery for colorectal cancer were enrolled in a quasi-experimental longitudinal study. In three hospitals, first a conventional care group (n = 250) was recruited, then the intervention was introduced, and finally the intervention group was recruited (n = 238). Patients’ trajectories of preparedness for surgery and recovery (Preparedness for Colorectal Cancer Surgery Questionnaire—PCSQ) health related quality of life (EORTC QLQ-C30) and distress (NCCS Distress Thermometer) were evaluated based on self-reported data at five time points, from pre-surgery to 6 months. Length of hospital stay and patients’ behavior in seeking health care pre- and post-surgery were extracted from patient records. Longitudinal structural equation models were used to test the hypothesized effects over time. Statistically significant positive effects were detected for two of the four PCSQ domains (patients searching for and making use of information, and making sense of the recovery) and for the role functioning domain of the EORTC QLQ-C30. Patients in the intervention group were also more likely to contact their assigned cancer “contact nurse” (a.k.a. nurse navigator) instead of contacting a nurse on duty at the ward or visiting the emergency department. In conclusion, the overall hypothesis was not confirmed. Further research is recommended on written and oral support tools to facilitate person-centred communication.

## Introduction

Person-centeredness is an emerging perspective in health care to acknowledge who the person in need of care is [1] and is increasingly considered a desired feature both as means for tailoring care to the individual’s needs and specific problems as well as the individual’s recourses and capacity to make sense of and handle challenges related to illness, treatments and care. Although there are generic aspects of person-centeredness with (potential) applicability across health care services and specializations, there is a need to contextualize person-centred interventions to specific patient populations and treatment paths [2]. This study focuses on the effectiveness of a clinical person-centred information and communication intervention to enhance patients’ preparedness for surgery and the recovery following colorectal cancer (CRC) surgery; which is one of the most common types of cancer worldwide affecting both men and women with a decreasing but still high mortality risk, with surgery being the primary treatment [3].

Person-centeredness could be positioned in the hermeneutics of self; the person being both suffering and capable [4, 5], and thus taking an ethical stance to the provision of care [6, 7]. This implies that the patient might become distressed when undergoing cancer treatment but also has resources to be prepared for understanding and responding to what is to come–if receiving appropriate support. Patients’ preparedness for surgery and recovery is a forward directed activity of what challenges and changes might come with cognitive (“to know”), emotional (“to feel”) and activity (“to be able to”) dimensions [8, 9]. Accordingly, communication is co-constructed interactively with interrelationships between meaning, self and context as well as scientific and experiential knowledge [10]. Further, person-centred communication in healthcare involves dimensions of health literacy [11]. Such enabling of the patient’s seeking for knowledge and learning [8] could be conceptualized as a transformation of experience [12]. This provides opportunities for clinical, person-centred communication interventions focusing on knowledge enablement in preparing patients for surgery and recovery. Although a person-centred approach focuses on individually tailored communication, this could be supported through the use of standardized tools [13]. Hence, person-centred communication is considered a complex intervention involving ways information is communicated [14, 15], both verbally and in writing, for example in patient education materials (PEM) [11].

Considering the previous sparse research on the effects of person-centred approaches to care with mixed results [16], we aligned with a more recent novel person-centred approach built around an ethical stance to engage patients and professionals dialoguing with each other instead of talking to or informing the patient [7, 17, 18]. Intervention studies in non-cancer populations suggest that such a person-centred approach added to conventional care has desirable outcomes, including: shortened hospital stay [19, 20] without increasing the risk for readmissions [20], improved discharge processes [21] and enhanced support for patients to manage recovery following hospitalization [22].

To minimize the physiological stress response associated with surgery, CRC care is marked by the ‘enhanced recovery after surgery’ (ERAS) procedures in relation to pre-, peri- and post-operative treatments [23–25]. These procedures in the context of CRC surgery involve a multimodal approach, including standardized pre-surgery information, optimized nutrition and pain management, and active mobilization leading to reduced hospital stay and complications [23, 26]. Although ERAS is positively evaluated in CRC care, patients may also report going through a transition from overcoming the surgery to recovering from it [27], including distress related to emotional, cognitive and behavioural dimensions [28]. Consequently, the ERAS focus on biomedical aspects and standardized patient information might not be sufficient and does not address person-centeredness [29].

Previous research has shown that CRC surgery and recovery has an impact on quality of life, health status and wellbeing [30], especially for patients with rectal cancer and receiving an ostomy [31]. However, the areas of concern for patients are only partly covered in existing instruments for measuring health-related quality of life (HRQOL) [32]. Following a diagnosis of gastrointestinal cancer, patients have been found to undergo experiential changes [30, 33, 34] corresponding to “recovery” as they regain control over biopsychosocial functions while striving to return to the preoperative level of independence in daily living and optimum well-being [35]. This implies that patients undergo recovery trajectories that are shaped by their health, quality of life and psychosocial factors [30]. Confidence of patients with CRC in being prepared to manage health related problems might predict HRQOL recovery trajectories independent of treatment or disease characteristics [30]. To facilitate the recovery process, patients with CRC need information and knowledge especially to be prepared for discharge, managing daily life at home and to understand the meaning of the cancer diagnosis [36].

A special challenge in CRC care communication is the timing of different CRC team members’ communication over time within interprofessional health care teams in order to enhance the patients’ recovery. However, the focus in cancer communication research has been patient–provider dyads with an emphasis on “sender–message–receiver” [15, 37]. Based on the premises of person-centeredness, it is important for communication to be contextualized in relation to the entire health care process surround the person’s unique illness trajectory. This becomes especially significant in relation to the individual’s emotional and social functioning, capabilities and wellbeing despite illness, which involves a person-centred approach [2, 38, 39]. Both the surgery and the cancer diagnosis are perceived to cause distress, while the person at the same time could be capable to handle consequences of both the cancer and its treatment. Then, how patients become prepared for surgery, discharge and recovery after surgery takes place at a time when the patient will have to make sense of their cancer diagnosis as well as the demands of the surgery, both biophysiologically and personally. This necessitates appropriate and timely patient information and communication by the entire health care team from diagnosis, during hospitalization and subsequent recovery after discharge [28, 40]. Hence, there is significant potential for person-centred interventions to prepare patients before CRC surgery and recovery following surgery.

Our previous exploration of information and communication in CRC care revealed several challenges [41–46]. Existing PEMs given to patients were characterized by low to adequate levels of readability, suitability and comprehensibility, which did not fully address the information needs of patients. The PEMs did not cover the whole recovery process and were particularly weak in relation to discharge, [44] and were further associated with a paternalistic discourse displaying problematic norms that potentially could be interpreted as power expressions [45]. Communication between patients and professionals during consultations was typically dominated or driven by the professionals. PEMs were seldom referred to and there was variability in the extent to which the subject and agenda for the consultation was introduced or not. Symptoms and recovery problems were infrequently discussed and most often introduced by patients [41]. Before surgery patients often received standardized information about perioperative routines and postoperative recovery procedures with communication characterized by provision of information [43]. Surgeons used strategies for actively communicating about bodily changes to enable patient understanding [42].

The aim of the study is to evaluate whether an intervention with a person-centred approach to information and communication for patients diagnosed with CRC undergoing surgery can improve the patients’ preparedness for surgery, discharge and recovery during six months following diagnosis and initial treatment.

## Methods

### Study design

The overall research design was a quasi-experimental longitudinal trial developed in accordance with the TREND statement for non-randomized controlled trials [47] (Registered at https://www.clinicaltrials.gov ID: NCT03587818). Two communication approaches were evaluated in three settings before and after the intervention was introduced; conventional care versus person-centred information and communication. Person-centred information and communication is a complex intervention [48, 49] requiring evidence based on both qualitative and quantitative research. Herein we report on the quantitative effect evaluation.

### Participants

**Fig 1:**
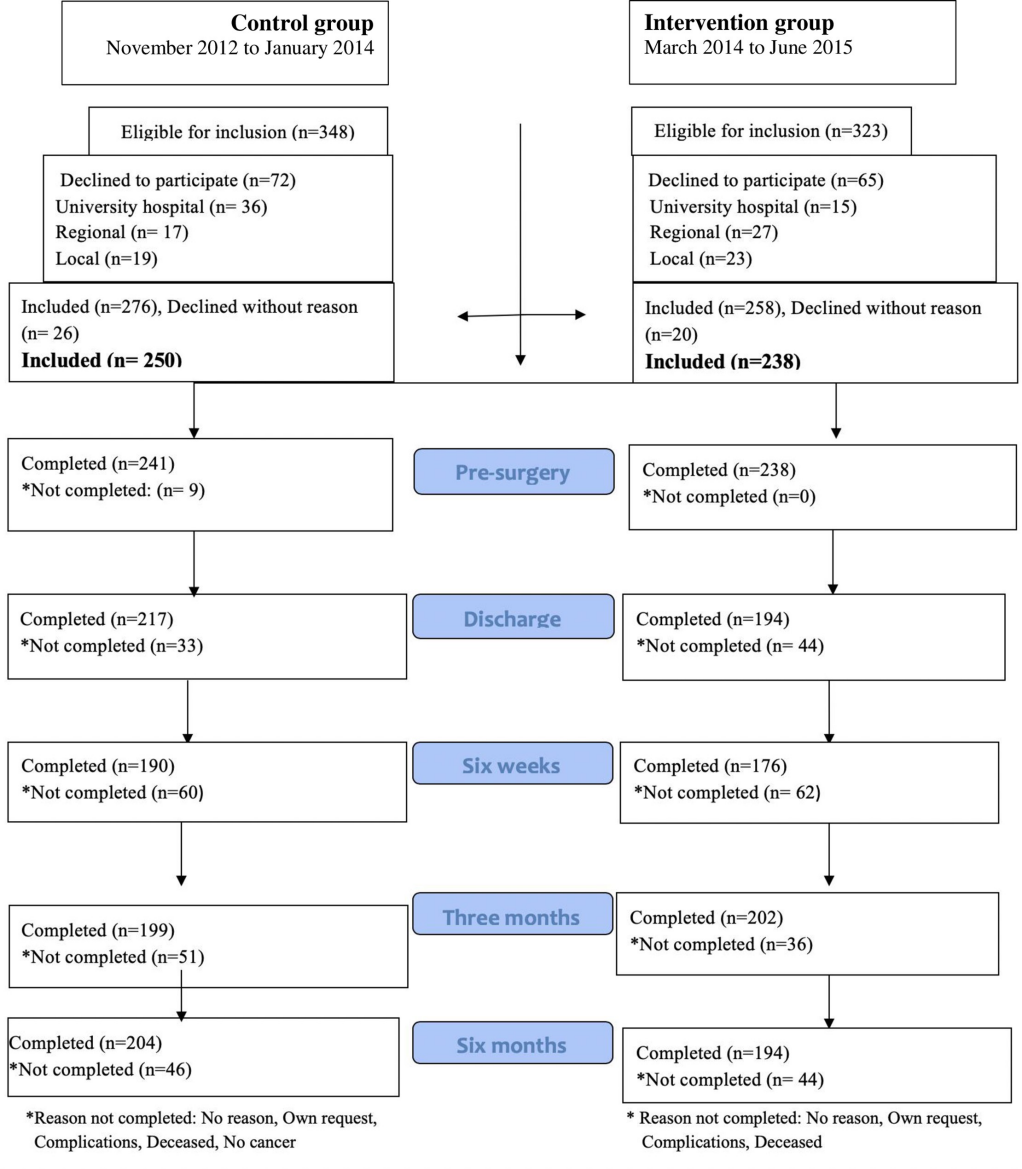
Study flow chart.

People undergoing elective surgery for cancer in the colon or rectum were eligible to participate. Exclusion criteria were receiving preoperative chemotherapy, long-term preoperative radiation, diagnosed metastasis, post-surgical diagnosis of benign tumours, undergoing emergency surgery, having reduced cognitive function, and lacking ability to communicate in Swedish. Patients were consecutively recruited from November 2012 to June 2015 from surgical departments at three hospitals in Sweden (including university, regional and local hospitals; public and private non-profit), see Fig 1.

### Conventional care and intervention

The study was implemented at CRC departments of three hospitals in Sweden: one university and one regional public hospital, and one local private not-for-profit hospital. The interprofessional health care team included surgeons and registered nurses (RNs), and assigned “cancer contact nurses” who were “the main point of contact, a resource for education, information, support and coordination of the clinical pathway for patients and their families” (p. 1303) [50] (like nurse navigators). The national policy is that all patients should be assigned a cancer contact nurse [51].

**Fig 2:**
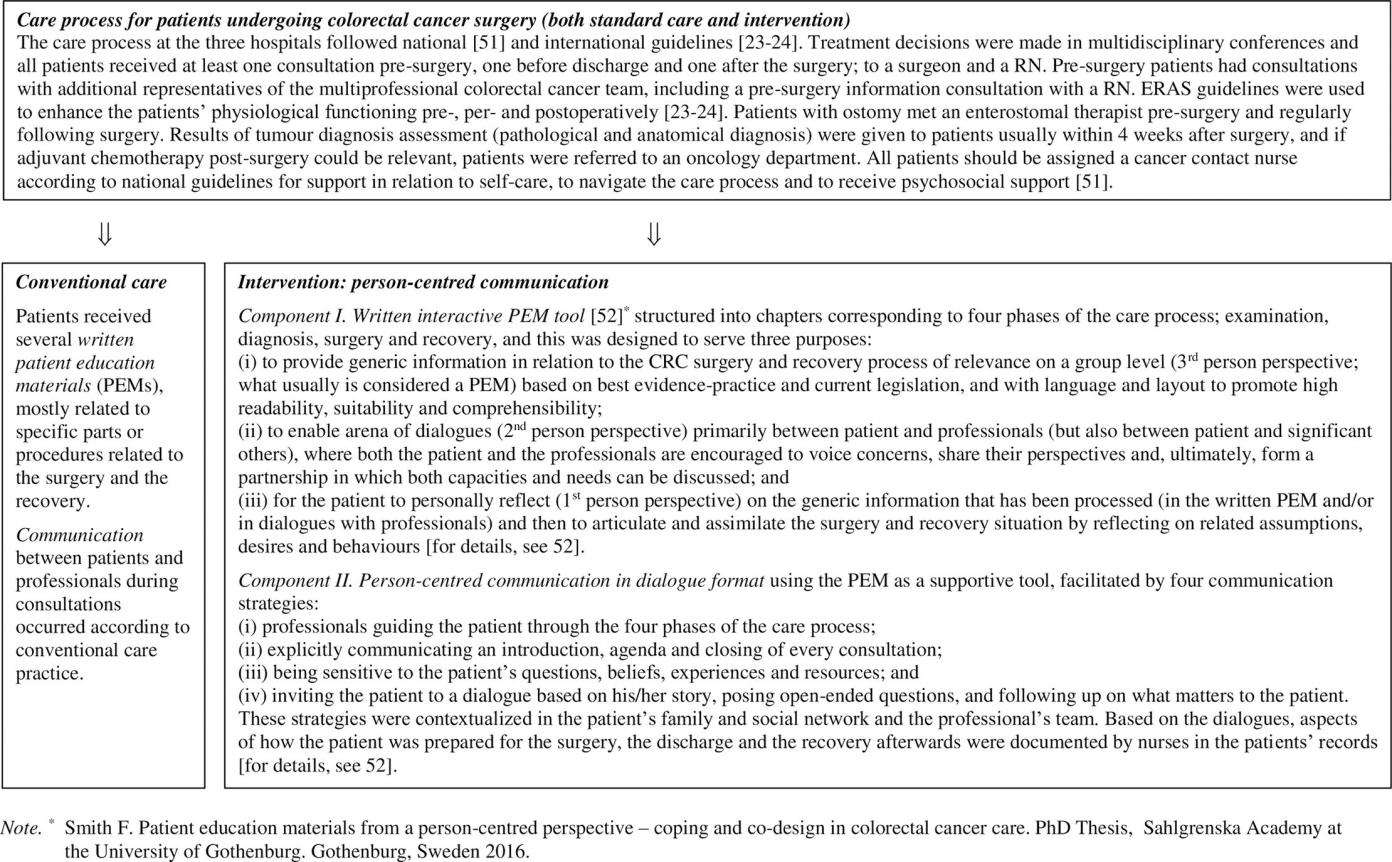
Conventional care and intervention as related to the overall care process.

**Fig 3:**
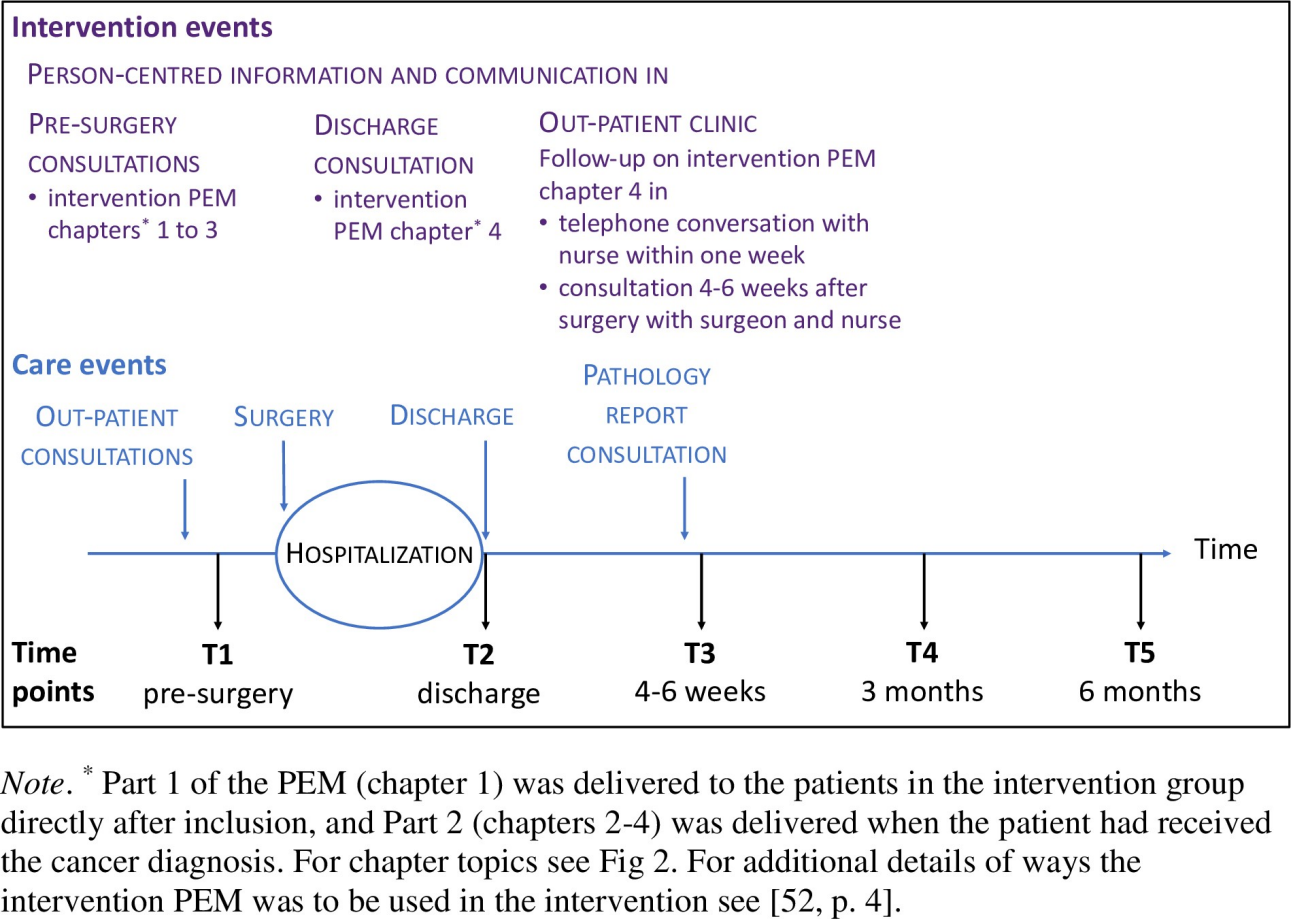
Specific intervention events as related to selected events in the care process for patients undergoing CRC surgery, and data collection time points.

Conventional care was mapped before patients were included in the study and the results thereof [41–46] informed the person-centred communication intervention, which was developed in collaboration between people who had undergone CRC surgery, professionals from CRC surgery clinics and researchers with expertise in patient education, person-centred care and CRC surgery. The intervention aimed to actively make use of a person-centred approach to support patients undergoing CRC surgery and enabling them to be prepared for surgery, discharge and recovery. This was accomplished through person-centred communication involving two components: 1) a novel written interactive PEM, and 2) an approach for professionals to facilitate person-centred communication [8, 39] during consultations. Details of the conventional care and the intervention components as related to the overall care process are displayed in Fig 2, and the specific intervention events as related to the care process are displayed in Fig 3.

To introduce the intervention, all participating professionals at the three hospitals respectively were invited to a two-hour workshop (in total 251 participants in 20 workshops), which included: a brief lecture on person-centeredness and person-centred communication, explanation of the two intervention components, and a tailored video concretizing and illustrating the two components of the intervention (produced by the research team). In addition, discussions and reflections were integrated throughout.

To ensure intervention fidelity facilitators (nurses and surgeons) at each hospital were assigned as primary contacts for the research team. During the ongoing intervention, repeated informal meetings were held with the nurse facilitators at the hospitals. In addition, an introductory intervention kit for self-directed learning was developed for professionals who did not take part in the introductory workshop. Two follow up workshops were held with nurse facilitators and representatives of cancer contact nurses (for details, see [52]).

### Hypothesis

Person-centred information and communication supported by an interactive PEM for patients undergoing CRC surgery will lead to improved preparedness for surgery and recovery during 6 months following surgery. Secondary outcomes were improved health related quality of life, reduced distress and decreased length of stay at hospital in relation to surgery, changed behaviour pertaining when and how to seek health care for recovery support.

### Outcomes: Data collection, instruments and clinical data

To enable a longitudinal analysis, questionnaires were delivered to patients at five time points: (1) before surgery (at pre-surgery information consultation), (2) at discharge after surgery (typically about 1 week after surgery), (3) four to six weeks after surgery, (4) three months after surgery and (5) six months after surgery (the time points as related to the intervention events are displayed in Fig 3). At the first and second time point participants received the questionnaire at the hospital and at all other time points a questionnaire was mailed. Participants returned the questionnaires in pre-stamped envelopes to a designated research nurse assistant at the three hospitals respectively.

The following self-reported outcome measures were used:

- The Longitudinal Preparedness for Colorectal Cancer Surgery Questionnaire (PCSQ) in Swedish measures preparedness for surgery and recovery over time in four domains: (i) searching for and making use of information (4 items), (ii) understanding and involvement in the care process (7 items), (iii) making sense of the recovery process (5 items), and (iv) support and access to medical care (7 items) [53]. The initial instrument development and evaluation of validity evidence has been reported by Carlsson et al [54]. The scale covers five specific time points: pre-surgery, discharge, 4–6 weeks, 3 months and 6 months post-surgery, and to ensure time-specific contextual applicability (i.e. pre-surgery, at discharge and during recovery), 14 of the 23 items have phase-specific wordings. There are four response alternatives for every item: (4) strongly agree, (3) agree somewhat, (2) disagree somewhat, and (1) totally disagree. Scores for each domain are obtained by calculating the average of its items when at least 50% of the items have valid responses. A total score is obtained by averaging the domain scores. Psychometric evaluation of the longitudinal PCSQ indicated that the measurement structure and parameters of the scale are consistent over time and that reliable scores could be obtained and compared across time-points [53]. The instrument has good internal consistency reliability (ordinal alpha > 0.93) in this sample for the four domains across all time points.
- EORTC QLQ-C30 version 3.0 (30 items) is a widely used measure of HRQOL for patients diagnosed with cancer and the Swedish version was used [55, 56]. Only the functional status scales and the “global health/quality of life (QoL)” scale were used: physical functioning (5 items), emotional functioning (4 items), role functioning (2 items), social functioning (2 items) and global health status/QoL (2 items) [57]. For the functional status scales, a 4-point Likert scale is used with response options ranging from (1) not at all, (2) a little, (3) quite a bit and (4) very much. The global health/QoL items have a 7-point Likert scale ranging from (1) very poor to (7) excellent. The EORTC QLQ-C30 scoring guidelines were followed to compute summary scores ranging from 0 to 100 when at least half of the items had valid responses. Internal consistency reliability was adequate in this study’s sample (ordinal alpha values were .74 and .82 for cognitive functioning and social functioning, respectively, and .91 or .93 for the other scales).
- The National Comprehensive Cancer Network (NCCS) Distress Thermometer (DT; Version 1.2013) is a widely used measure to detect clinically significant distress in patients [58]. The one item thermometer scale in a Swedish version [59] was used, which consists of a visual analogue scale ranging from 0 (no distress) to 10 (extreme distress), where participants are asked to indicate how much distress they have been experiencing the past week including today.

The pre-surgery questionnaire included additional questions about individual characteristics: sex, age, social background and living conditions, and occupational situation. Participants in the intervention group responded to a question whether they had received the intervention PEM (yes/no) and reported on how useful they perceived the PEM based on a 4-point Likert scale ranging from (1) not at all to (7) very much (a figure with the covers of the PEM were included).

The following clinical information was retrospectively obtained from patients’ medical records: cancer diagnosis according to ICD-10 [60], ASA classification pertaining medical fitness before surgery as assessed by a responsible anaesthesiologist [61], type of surgery [62], tumour staging [63], presence of adjuvant therapy post-surgery, length of stay (number of days hospitalized in relation to the CRC surgery), number of phone calls to cancer contact nurse pre surgery and after discharge, number visits to emergency room after discharge, and number of hospital readmissions.

Dedicated research nurses were at the three hospitals respectively assigned to monitor patient inclusion and to deliver the questionnaires to the individual patients at correct time points. For advice and information about their participant, patients were instructed to contact the research nurses or one of the researchers. Data entry was performed twice and where a difference in data was identified a third check to the original questionnaire was performed.

### Sample size

A Montecarlo simulation of a linear trajectory model with 5 time points was conducted to project the required sample size. Given that the intervention is designed to directly target the primary endpoint, we anticipated a large effect size, corresponding with a standardized difference of 0.1 between the slopes of the intervention and control groups. Based on these premises and a two-sided test with a type I error rate of 0.05, a sample size of 250 in each group would be required.

### Assignment method

A before and after design was used. No blinding was applied. Control group participants were recruited consecutively between November 2012 and January 2014. Introducing the intervention to the clinicians followed this. Intervention group participants were subsequently recruited from March 2014 to June 2015. Although data collection for the control group continued during the first 4 months of the intervention recruitment period, there were no scheduled interactions between control group participants and health care professionals during that period. Still, interactions initiated by patients took place.

### Analyses

Descriptive statistics (means and standard deviations for continuous variables, and frequencies and percentages for categorical variables) were used to characterize the groups. Statistical significance of differences between the groups at time point 1 was ascertained using the Chi-Square test for dichotomous and nominal variables, Mann-Whitney U test for ordered categorical variables, and T-test for continuous variables. The same statistical tests were used to compare the sample (age, type of cancer, tumor stage, ASA class) to the national CRC population data from the Swedish Colorectal Cancer Registry.

Longitudinal structural equation models (SEM) were used to test the hypothesized intervention effects on the self-reported outcome variables (preparedness domains, functional status domains, global health/quality of life and distress) by comparing the intercepts and slopes of the trajectories of each outcome variable across intervention and control groups, which were based on intention to treat [64, 65]. Following established guidelines for longitudinal SEMs, as described by Heck and Thomas (2015), the models were specified with two latent factors representing the intercept (outcome score at time point 1) and the slope (change in outcome score over time). All loadings of the latent factor representing the intercept were fixed at 0. For the latent factor representing the slope, the loadings representing time points 1 and 3 were fixed at 0 and 1 (for purposes of model identification) and the remaining loadings were freely estimated thereby allowing for differences in shape of the trajectories between groups. The intercept and the slope were regressed on the grouping variable (control versus intervention) and the following covariates to adjust for the possibility of confounding: age, sex, ostomy, presence of reoperations, adjuvant therapy, hospital, type of cancer, ASA class, type of surgery and readmissions. A difference between the two groups at time point 1 is indicated by a statistically significant regression parameter estimate for the grouping variable on the intercept (*p* <0.05). A difference in the average change over the six-month period (i.e., the intervention effect) is indicated by a statistically significant regression parameter estimate for the grouping variable on the slope. Full information maximum likelihood (FIML) and robust estimation (MLR) were applied using the Mplus version 8.0 [66]. Evaluation of model fit was informed by comparisons of observed and predicted trajectories, a chi-square significance test, and the following guidelines by Hu and Bentler who suggested that a root mean square error or approximation (RMSEA) around 0.06 (or less) and a comparative fix index (CFI) close to 0.95 is indicative of adequate fit [67].

For the outcome variables that did not vary over time, a one-way ANOVA was conducted to evaluate the difference in length of stay (using a log transformation) between intervention and control groups. In addition, multinomial logistic regression was used to evaluate between-group differences in variables measuring contacts before surgery and after surgery (collapsed into ordinal categories) while controlling for the same covariates as listed above for the longitudinal SEMs.

The dropout rate was 20% over the six-month period (see Fig 1). The percentages of missing data (including dropouts) for variables included in the analyses of outcomes were 4.9, 19.7, 30.5, 21.9, and 22.9 for the time-varying outcomes at time points 1 to 5, respectively, 2.5% for the time-invariant outcomes, 1.5% for the covariates (total % missing data = 18.4%). Mean imputation was used in computing subscales scores to accommodate missingness on EORTC and PCSQ items when at least 50% of the items had complete data (based on the scoring instructions for these instruments). Multiple imputation (MI) was used to accommodate missing data on the covariates (1.5% imputed data) and non-time varying outcome variables (2.5% imputed data) [68]. The MPlus 8.0 software was used to implement MI of 50 datasets using the Markov chain Monte Carlo method and maximum likelihood estimation, and to subsequently obtain pooled estimates. Categorical variables were dummy-coded prior to MI and all available variables were included as covariates to improve accuracy of imputation [69]. Subsequently, the longitudinal SEMs were conducted using full information maximum likelihood (FIML) to accommodate longitudinal missing data (dropouts) for time-varying outcome variables (EORTC and PCSQ domains and DT). This was done to ensure compatibility between the imputation model and the analysis model [70]. Both FIML and MI have similar assumptions and assume missingness to be at random (MAR), but not completely at random [71]. Sensitivity analyses on select outcomes confirmed that the above combined FIML and MI approach yielded results that were nearly equivalent to those based on the exclusive use MI.

### Ethical considerations

The study was approved by the Regional Ethical Review Board in Gothenburg (Dnr 536–12) and the project conformed to the principles in the Declaration of Helsinki. Eligible patients at the outpatient departments were initially informed in person by their responsible surgeon or RN about the study, and those who were interested received written information. Assigned RNs provided follow up on the written information and provided opportunity for questions about participating in the study before written informed consent was obtained. The voluntary aspect of participation was emphasized and there were patients who initially signed the consent form and never participated, those who participated only one or a few time points and others who discontinued at all time points.

Based on the researchers’ knowledge, it was anticipated that patients would experience challenges at the time of discharge (time point 2). Therefore, special attention was given to minimize respondent burden [72] at time point 2 by reducing the length of the questionnaire (several of the EORTC subscales were not included).

## Results

In total, 488 patients eligible for CRC elective surgery were included; 250 in the control group and 238 in the intervention group (see flowchart, Fig 1). The control and intervention groups were similar with regard to demographic, diagnostic and treatment variables, with one exception; laparoscopic surgery was more frequent in the intervention group than in the control group (Table 1). The sample in this study (control and intervention groups) was comparable to the national population of patients undergoing CRC surgery with respect to sex, tumour stage and presence of reoperations. However, there were statistically significant differences in the sample as compared to the national population in: age (the sample was younger; mean age 69 vs 73 years, *p* = <0.001), type of cancer (higher proportion of patients with rectal cancer; 39.6% vs 29.8%, *p* = <0.001), ASA class (higher proportion of patients with lower ASA classes, *p* = <0.001) and ostomy (higher proportion of patients with an ostomy; 39.1% vs 31.8%, *p* = 0.001).

**Table 1:** Characteristics of the participants.

Variables (% missing in control group/intervention group)	Control group (n = 250)	Intervention group (n = 238)	P-value
***Demographics***			
Men/Women (0.0%/0.0%)	56.8/43.2	54.6/45.4	0.694
Age (mean; SD) (0.0%/2.1%) ^1^	(67.4; 11.6)	(69.0; 10.8)	0.116
Marital status (5.6%/0.0%)			0.415
Married	62.7	61.8	
Unmarried	27.5	24.8	
Widow/widower	9.7	13.4	
Household (6.0%/1.3%)			0.260
Cohabiting	74.5	68.9	
Living apart	4.7	3.8	
Single	20.9	27.2	
Living arrangement (5.2%/0.0%)			0.698
Villa	47.3	42	
Condominium	25.3	29	
Rental apartment	25.7	27.3	
Nursing home	1.7	1.7	
Native language (12.4%/8.8%)			0.689
Swedish	86.8	88.5	
Other	13.2	11.5	
Country of birth (4.8%/0.0%)			0.894
Sweden	86.6	85.7	
Other	13.4	14.3	
Parents’ country of birth (4.8%/0.0%)			0.732
Both Sweden	81.5	82.4	
One Sweden, one other	3.8	2.5	
Both other	14.7	15.1	
Education (5.6%/0.4%)			0.244
Elementary school	27.5	26.6	
High school	22	24.1	
University/college	34.7	30	
Residential college	4.7	2.5	
Other	11.0	16.9	
Employment (4.8%/0.0%)			0.202
Working ^2^	36.6	27.7	
Studying	0.4	0	
Seeking employment	0.8	1.3	
Retired	61.3	70.6	
Other employment	0.8	0.4	
***Diagnoses***			
Type of cancer (0.0%/4.6%)			0.519
Colon cancer	58.8	62.1	
Rectal cancer	41.2	37.9	
Tumour stage (6.4%/6.3%)			0.091
I/II;	33.3	28.7	
III	61.5	61	
IV	5.1	10.3	
ASA Class (0.4%/0.8%)			0.348
ASA 1; healthy	16.9	15.3	
ASA 2; mild systemic disease	61	69.1	
ASA 3; severe systemic disease	20.9	15.3	
ASA 4; constant severe systemic disease that is a constant threat to life	1.2	0.4	
Presence of cancer history before the surgery (0.8%/0.0%)			0.856
Yes	13.3	14.3	
No	86.7	85.7	
Presence of other cancer diagnosis in addition to the CRC (0.8%/0.0%)			0.266
Yes	4.8	2.5	
No	95.2	97.5	
***Treatments and care***			
Hospital (0.0%/0.0%)			0.073
I	49.6	59.2	
II	22.0	15.5	
III	28.4	25.2	
Type of surgery (0.4%/0.0%)			0.659
Rectal resection	24.9	22.7	
Rectal ablation with perianal wound, or larger resection of colon with ostomy	15.7	13.9	
Rectal-sigmoid resection, or right hemicolectomy	59.4	63.4	
Laparoscopic surgery (0.4%/0.0%)			0.001
Yes	21.7	35.7	
No	78.3	64.3	
Ostomy (0.0%/0.8%)			0.547
Loopileostomy	22,4	18,5	
Colostomy	18,0	18,5	
Presence of reoperation(s) (1.6%/0.8%)			0.842
Yes	8.5	7.6	
No	91.5	92.4	
In contact with cancer contact nurse (5.3/4.6%)	82.2	84.2	0.562
Number of readmissions (16.4%/2.1%)			0.728
0	74.6	76.4	
1	19.1	16.7	
2	4.8	4.7	
≥3	1.5	2.2	
Adjuvant chemotherapy (2.4%/0.8%)			0.715
Yes	30.3	28.4	
No	69.7	71.6	

Notes.

^1^Range: control group 32–90 years and intervention group 37–92 years.

^2^ Including on sick leave.

In the intervention group, 87.0% were hospitalized in one of the intervention wards, whereas 13.0% received care at other wards where the professionals not to the same extent had introduced to the intervention. Nonetheless, 95.4% (0.4% missing) of all participants in the intervention group reported receiving the intervention PEM tool and, of these, 97.7% reported the PEM was quite a bit or very useful in conversations with professionals (and 2.4% not at all or a little useful; 11.3% missing). In the control group 88.9% (6.4% missing) and in the intervention group 95.4% (0.0% missing) knew who their cancer contact nurse was, and 82.2% (7.6% missing) in the control group and 84.1% (1.7% missing) in the intervention group had been in contact with their cancer contact nurse.

### Preparedness for surgery and recovery

**Fig 4:**
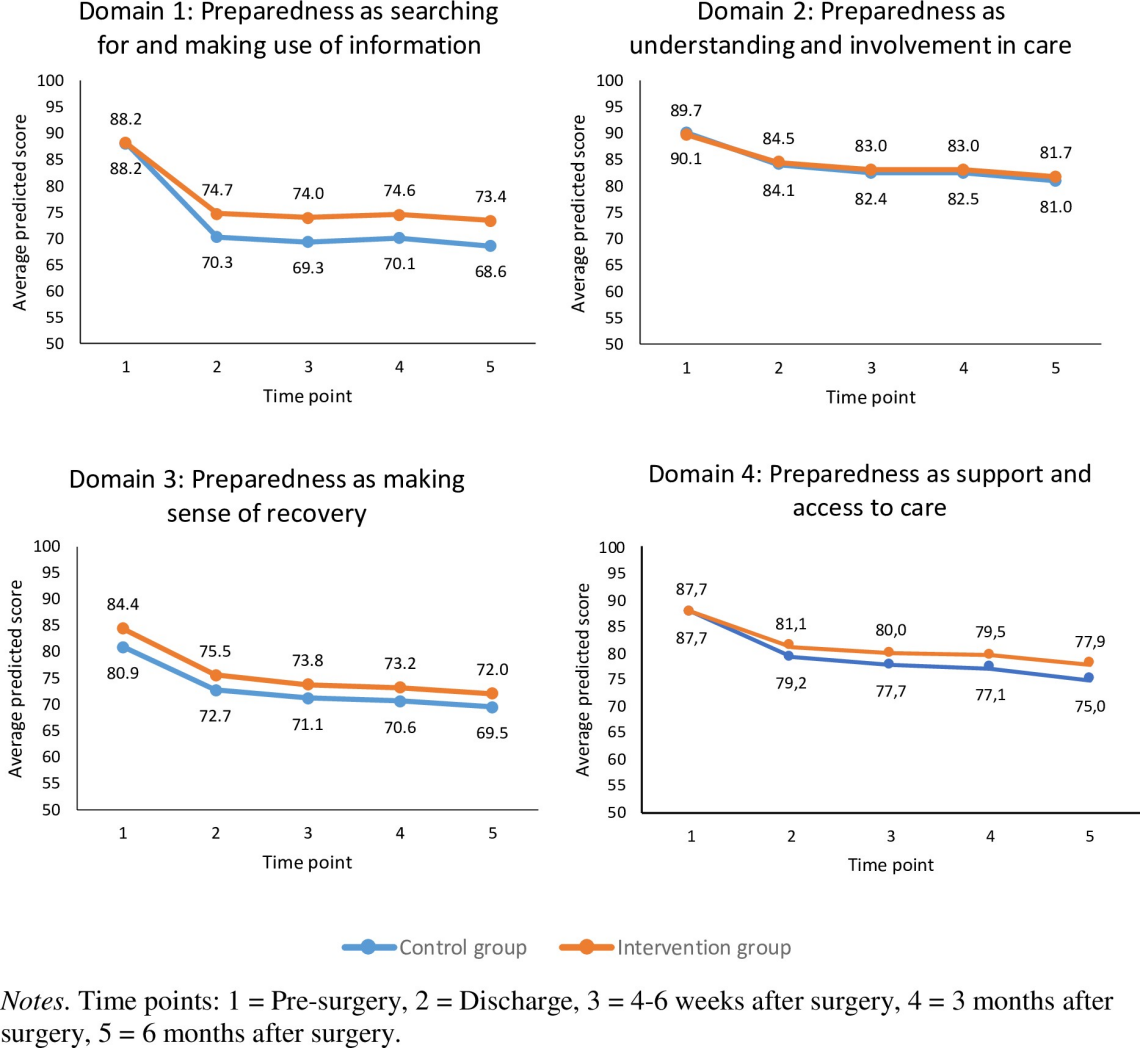
Trajectories of patient preparedness for surgery and recovery.

The SEMs for testing the hypothesized intervention effects on preparedness resulted in adequate model fit (Table 2). There was a statistically significant decline in each of the preparedness domains as assessed with the PCSQ over the six-month time period, indicating that patients (control and intervention groups) initially felt quite prepared before surgery but less prepared after surgery (see Fig 4). In addition, relative to the control group, patients in the intervention group reported less decline in the domain “searching for and making use of information” (slopes for control and intervention groups were -18.8 and -14.8, respectively, *p* = 0.01). Relative to the intervention group, the control group participants reported lower scores for the domain “making sense of the recovery process” at time point 1 pre-surgery (intercepts were 80.9 and 84.4 in the control and intervention groups, *p* = 0.04) but no difference was detected in the slope of the trajectory. There were no statistically significant differences in intercepts or slopes between the two groups for “understanding and involvement in the care process” and “support and access to medical care” (Fig 4; Table 2).

**Table 2:** Structural equation modelling results.

	Preparedness domains (PSCQ)				Global health/QOL and functional status domains (EORTC)						Distress thermometer Est. (*p*)
	Domain 1 Est.(*95% CI)*)	Domain 2 Est. (*p*)	Domain 3 Est. (*p*)	Domain 4 Est. (*p*)	QOL^1^ Est. (*p*)	EMOTION Est. (*p*)	PHYSICAL^1^ Est. (*p*)	ROLE^1^ Est. (*p*)	COGNITIVE^1^ Est. (*p*)	SOCIAL^1^ Est. (*p*)	
**Regression on intercept**^2^											
Intercept	88.2 (86.3:90.1) *p <* 0.001	90.1 (88.5:91.7) *p <* 0.001	80.1 (78.4:83.3) *p <* 0.001	87.7 (85.9:89.6) *p <* 0.001	71.2 (68.2:74.2) *p <* 0.001	73.6 (71.2:76) *p <* 0.001	86.3 (84.4:88.3) *p <* 0.001	83.3 (79.5:87.1) *p <* 0.001	87.1 (84.9:89.4) *p <* 0.001	86.7 (84:89.3) *p <* 0.001	3.0 (2.7:3.2) *p <* 0.001
Intervention^3^	0.0 (-2.6:2.6) *p* = 0.99	-0.4 (-2.7:2.0) *p* = 0.76	3.5 (0.2:6.8) *p* = 0.04	-0.1 (-2.7:2.5) *p* = 0.97	-2.4 (-6.8:2.0) *p* = 0.28	-1.5 (-4.9:2) *p* = 0.41	-1.8 (-4.7:1.2) *p* = 0.25	-4.2 (-9.1:0.7) *p* = 0.09	-0.9 (-4.3:2.5) *p* = 0.61	-4.0 (-8.1:0.2) *p* = 0.06	0.3 (-0.1:0.6) *p* = 0.15
**Regression on slope**^2^											
Slope	-18.8 (16.3:21.4) *p <* 0.001	-7.6 (-9.9:-5.4) *p <* 0.001	-9.7 (-12.5:-7.0) *p <* 0.001	-10.0 (-12.3:-7.7) *p <* 0.001	-0.3 (-3.7:3.1) *p* = 0.88	7.5 (5:9.9) *p <* 0.001	-11.5 (-14.2:-8.9) *p <* 0.001	-17.5 (-22.2:-12.9) *p <* 0.001	-1.1 (-2.6:0.5) *p* = 0.18	-6.3 (-9.9:-2.6) *p <* 0.001	-0.0 (-0.5:0.0) (0.04)
Intervention effect^3^	4.7 (1.3:8.1) *p* = 0.01	0.9 (-1.8:3.7) *p* = 0.51	-0.9 (-4.4:2.7) *p* = 0.64	2.3 (-0.5:5.1) *p* = 0.11	2.6 (-2.4:7.7) *p* = 0.30	0.3 (-2.4:3) *p* = 0.81	2.6 (-0.9:6.2) *p* = 0.15	9.6 (2.2:16.9) *p* = 0.01	0.6 (-1.2:2.5) *p* = 0.50	3.9 (-0.9:8.8) *p* = 0.11	-0.07 (-0.3:0.1) *p* = 0.45
**Model fit**											
Χ^2^ (Df)	95.5 (46)	103.8 (46)	80.0 (46)	88.2 (46)	84.9 (29)	60.1 (29)	74.9 (29)	60.1 (29)	27.1 (29)	54.7 (29)	54.8
RMSEA	0.05	0.05	0.04	0.04	0.06	0.03	0.06	0.05	0.00	0.04	0.02
CFI	0.94	0.93	0.96	0.95	0.90	0.98	0.94	0.93	1.00	0.95	0.98

*Notes*. Est. = parameter estimate. RMSEA = root mean square error of approximation. CFI = comparative fit index. Slope = change from time point 1 to 3. Intercept = score at time point 1.

^1^ EORTC subscale not administered at time point 2.

^2^ Controlling for age, sex, ostomy, presence of reoperations, adjuvant therapy, hospital, type of cancer, ASA class, type of surgery and readmissions.

^3^ 0 = control group, 1 = intervention group.

### Health-related quality of life and distress

**Fig 5:**
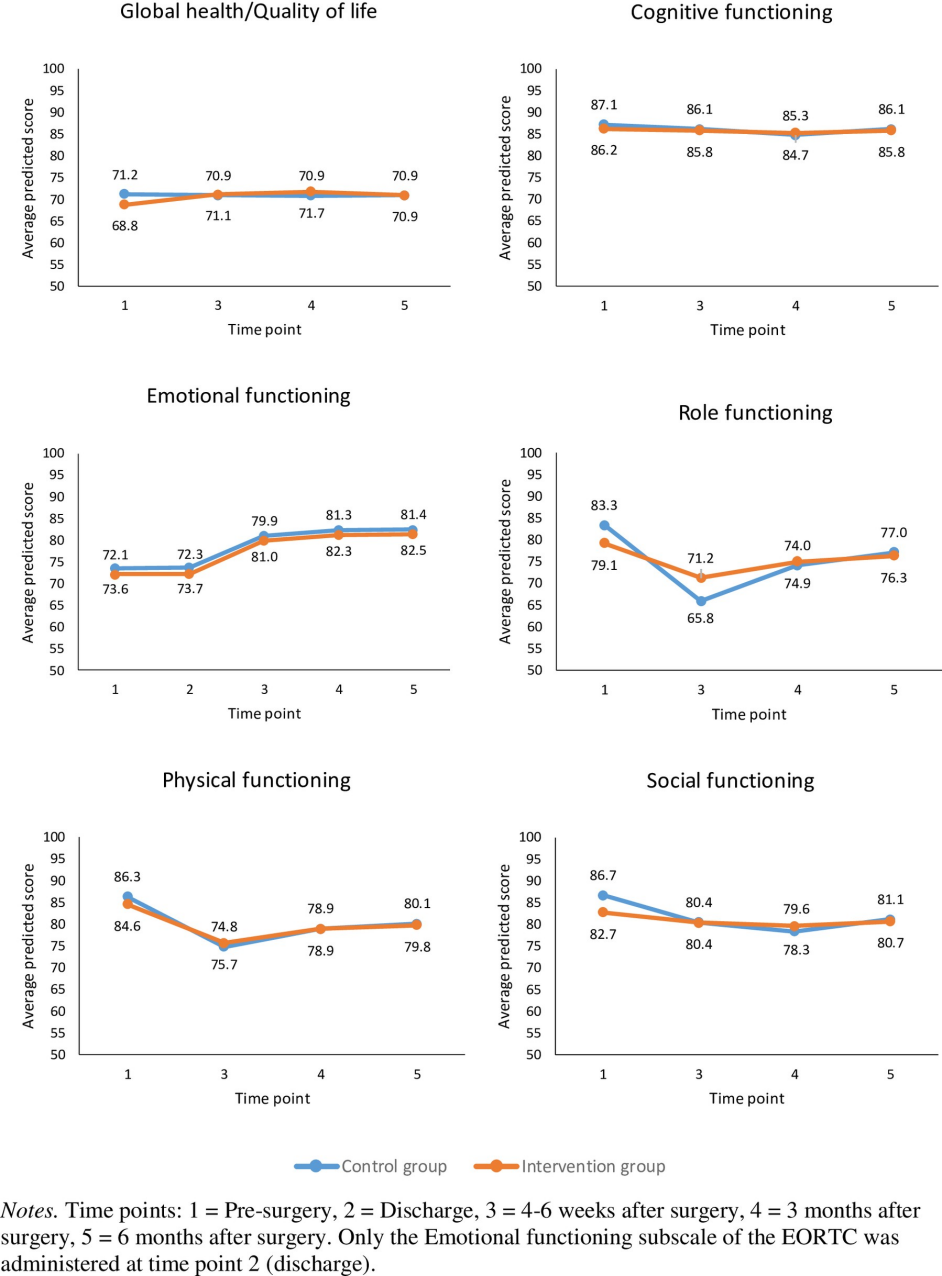
Trajectories of global health/quality of life, cognitive, emotional, role, social, and physical functioning.

**Fig 6:**
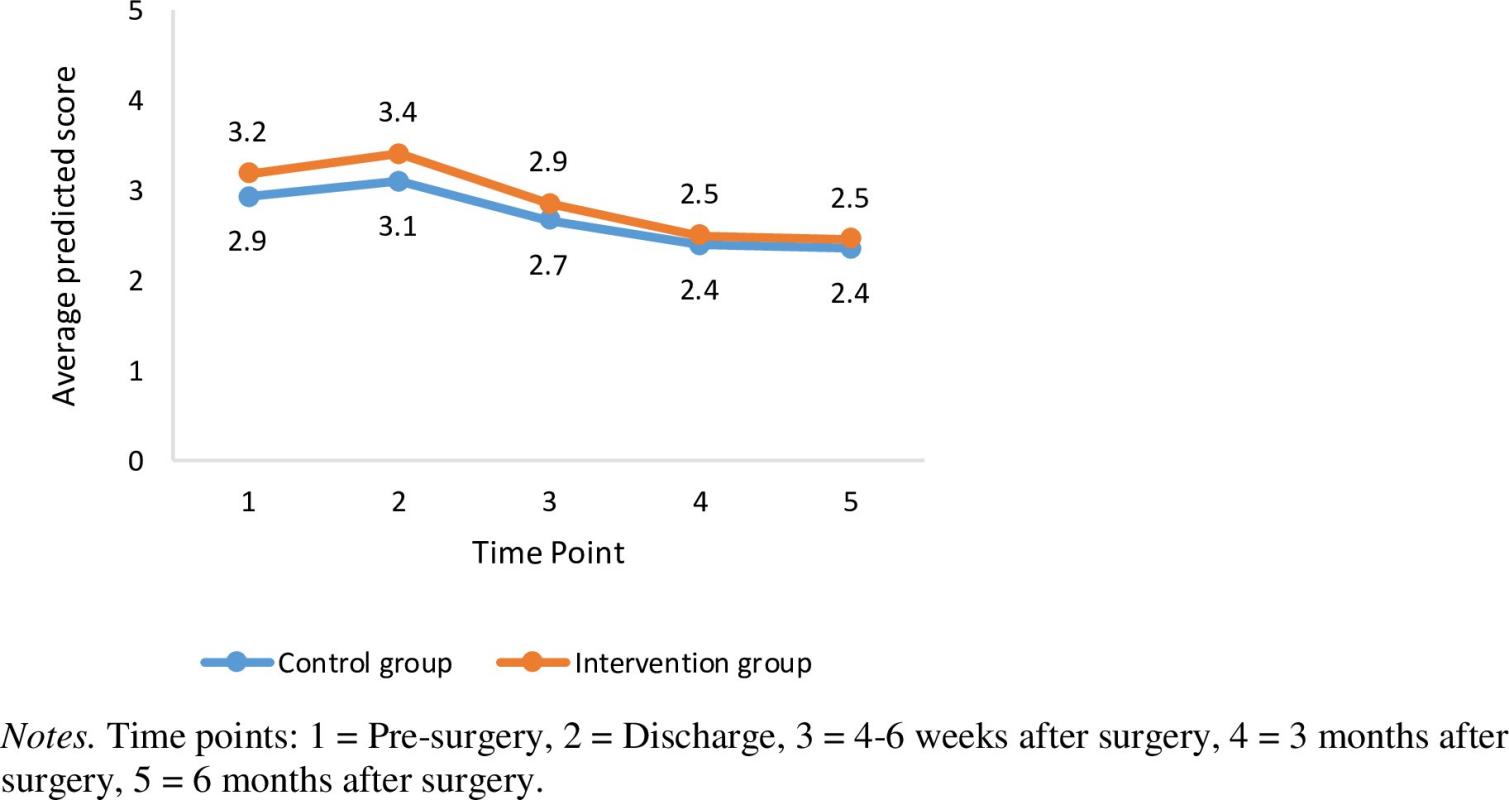
Trajectories of distress.

The SEM focusing on HRQOL domains (EORTC-QLQ-C30) and distress (DT) resulted in adequate model fit (Table 2). Based on the EORTC-QLQ-C30, patients reported an improvement in their emotion function from pre- to post-surgery over the six-month period (average slope = 7.6); there were no differences in the intercept and slope between the two groups (Fig 5). However, patients reported a decline in their physical function (average slope = -10.2) and, to a lesser extent, their social function (average slope = -4.3), with no statistically significant differences in the intercepts or slopes between the two groups. Patients also reported a decline in their role function; however, there was a statistically significant difference in the slopes between the two groups (-17.5 versus -7.9 in the control and intervention groups, *p* = 0.01). Patient’s reported general health and cognitive function remained stable over the six-month period with no group differences being detected. With respect distress (DT), the results indicate an initial increase in distress at the time of discharge followed by decreased distress overtime thereafter (Fig 6). The average slope was -0.30 with no statistically significant differences detected between the two groups, see Table 2.

### Length of stay

The length of stay patients who were hospitalized in relation to surgery was 8.8 days (median = 8.0) for the control group compared with 8.0 days (median = 7.0) in the intervention group (N = 488, *p* = 0.033, based on the logarithm of length of stay). In comparison, post-hoc tests based on a one-way ANOVA indicate that differences with the national population of patients undergoing CRC surgery (N = 7718, mean = 8.6, median = 7) were not statistically significant (*p* = 0.228 and 0.146 for control and intervention groups).

### Patient behaviour in seeking health care

Pre-surgery, patients in the intervention group were more likely than patients in the control group to have visited a cancer contact nurse once (56.0% versus 39.6%, OR = 8.8) or multiple times (38.5% versus 30.0%, OR = 8.0), or to have phoned a cancer contact nurse at least once (34.2% versus 23.6%, OR = 1.9) or twice (14.4% versus 10.8%, OR = 2.2) (see Table 3). After discharge, patients in the intervention group were also more likely to have phoned the assigned cancer contact nurse once (41.9% versus 26.2%, OR = 2.8) or more than twice (15.8% versus 7.6%, OR = 5.1). Conversely, intervention group patients visited the outpatient surgery department without the assigned cancer contact nurse less frequently (71.2% had one or more visits in the intervention group, versus 95.4%% in the control group; see Table 3 for details) and were also less likely to have phoned the nurse on duty at the surgical ward multiple times (2.6% versus 18.0%, OR = 15.4). Visits to the outpatient surgery department were slightly more frequent for the intervention group, compared to the control group (97% versus 93% had at least one visit; see Table 3 for details). However, visits to emergency departments were markedly less frequent, with only 3.8% and 2.1% of patients in the intervention group having visited the emergency department once or multiple times, as compared to 15.0% and 5.9% in the control group (ORs = 6.7 and 5.7, respectively).

**Table 3:** Patients’ behaviour for seeking healthcare (N = 488).

	Estimated Percentages		Intervention vs Control group	
Outcome variables	Control group	Intervention group	Odds ratio (95% CI)	p-value
**Contacts before surgery**				
To the assigned cancer contact nurse number of visits ^i^				
• 0	30.4	5.5	--	
• 1	39.6	56.0	8.8 (5.0–15.3)	<0.001
• ≥2	30.0	38.5	8.0 (4.5–14.3)	<0.001
- number of phone calls ^ii^				
0	56.0	44.1	--	
1	23.6	34.2	1.9 (1.3–2.8)	0.006
2	10.8	14.4	2.2 (1.3–3.7)	0.018
≥3	9.6	7.2	1.3 (0.7–2.6)	0.514
**Contacts after discharge**				
To the assigned cancer contact nurse - number of phone calls ^iii^				
• 0	51.6	28.6	--	
• 1	26.2	41.9	2.8 (1.9–4.1)	<0.001
• 2	14.9	13.7	1.7 (1.0–2.8)	0.074
• ≥3	7.6	15.8	5.1 (2.6–9.9)	<0.001
To the outpatient surgery departmentnumber of visits without the assigned cancer contact nurse ^iv^				
• 0	4.6	28.8	--	
• 1	72.6	42.0	14.1 (7.6–22.3)	<0.001
• 2	16.2	13.6	17.8 (8.3–38.1)	<0.001
• ≥3	7.4	15.7	9.1 (3.9–21.1)	<0.001
- number of visits within 6 weeks after discharge ^v^				
• 0	7.0	3.0	--	
• 1	89.0	92.4	3.1 (1.4–7.0)	0.022
• ≥2	4.0	4.7	4.0 (1.4–11.8)	0.034
Nurse on duty at the surgical ward - number of phone calls ^vi^				
• 0	63.7	78.2	--	
• 1	18.4	19.2	1.7 (1.0–2.9)	0.066
• ≥2	18.0	2.6	15.4 (6.1–38.6)	<0.001
Emergency department- number of visits ^vii^				
• 0	79.2	94.1	--	
• 1	15.0	3.8	6.7 (3.0–14.7)	<0.001
• ≥2	5.9	2.1	5.7 (1.9–17.1)	0.010

*Notes*. Percentages are estimated using multiple imputation. Odds ratios and *p*-values are based on multinomial logistic regression analysis controlling for age, sex, ostomy, presence of reoperations, adjuvant therapy, hospital, type of cancer, ASA class, type of surgery and readmissions.

^i^ Not controlling for hospital, due to sparse cell sizes.

^ii^ Range control group 0–4, intervention group 0–6 phone calls.

^iii^ Range control group 0–9, intervention group 0–22 phone calls.

^iv^ Range control group 0–26, intervention group 0–22 visits.

^v^ Range control and intervention groups 0–11 visits.

^vi^ Range control and intervention groups 0–11 phone calls.

^vii^ Range control group 0–4, intervention group 0–5 visits.

^iv, vi, vii^ Odds ratios are inversed to facilitate interpretation.

## Discussion

In this project, person-centred information and communication was contextualized and applied to patients undergoing CRC surgery, a specific challenging treatment involving both a recent diagnosis of cancer and undergoing a major surgery. The results show that the CRC surgery (and possibly also the cancer diagnosis itself and other treatments and care related) has a severe impact on patients’ recovery since they did not regain their pre-surgery levels in physical and role function during the 6-month study period. Patients were less prepared for discharge and the recovery following surgery than they were prepared for the surgery. However, patients became less distressed over the 6 months following surgery.

The major hypothesis was not supported as patients’ preparedness for surgery and recovery following surgery was not improved in the intervention group as compared to the control group for all four domains of the PCSQ; significant effects were only detected for patients searching for and making use of information, and for making sense of the recovery. Impact of the intervention was also detected for several secondary outcomes: patients in the intervention group had better role functioning, shorter length of stay in hospital following surgery, and they contacted their assigned cancer contact nurse instead of contacting a nurse on duty at the ward or visiting the emergency department.

These results suggest the intervention is associated with more active patients who were prepared to search for and make use of information, and make sense of the recovery (Fig 4), and who contacted the appropriate healthcare professional instead of the surgical ward or emergency department (Table 2). In this way, the intervention enabled person-centeredness in terms of supporting patients’ personal capability with acquired knowledge and ability to take appropriate action (Cf. e.g. [5, 8]). This capability dimension was especially highlighted in the newly developed PEM, which was designed to support patients coping over time in relation to the CRC surgery (for details, see [69]). However, considering that the intervention was complex and included two interrelated components (a novel written interactive PEM tool and person-centred communication in dialogue format) raises questions about the extent to which both components were implemented. In this study, patients reported the PEM to be useful. In addition, a process evaluation of the intervention revealed that professionals involved in delivering the intervention valued both of the intervention components, and in particular the interactive PEM tool [52]. However, the actual communication in a sample of audio-taped consultation conversations from the intervention was variously characterized by professionals as either *talking with* or *taking to* the patient, which indicates that the person-centred communication component of the intervention was not consistently applied [73]. Further, areas of ambiguity were disclosed related to the professionals’ own practice, contextual conditions and the planned intention of the intervention [52]. Hence, the overall results of the project reiterate the well-known challenges in changing communication practices in healthcare [14, 15]. Consequently, the effect observed in our study may be explained by any of the following: (i) the novel written interactive PEM tool that was delivered to all participants, (ii) person-centred communication in dialogue format that was applied in an unknown proportion of consultations, (iii) support from the assigned cancer contact nurses and (iv) other possibly unobserved factors. Hence, securing the implementation of both intervention components, and providing additional facilitation to support professionals to integrate and practice the intervention is suggested in future research. An optimized time frame in between the inclusion of participants in the control and the intervention groups to allow sufficient time for professionals to integrate the intervention in their practice should be considered. In relation to the study findings from the process evaluations indicating the intervention supported patients’ personal capability, it might be the two domains of the PCSQ with significant effects that could be influenced by the intervention. Probably, also conventional care supports the patients’ understanding and involvement in care, and their support and access to care.

The decreased length of stay in hospital associated with the intervention group should be viewed in relation to the already shortened length of hospitalization obtained through the ERAS procedures [23–25]. While ERAS is designed to minimize the physiological stress-response associated with surgery, our results point to the possible benefits of person-centred information and communication. Hence, CRC care might benefit from adding person-centeredness to the well-established concepts of ERAS and personalized medicine; of which the latter is a biomedical concept for optimizing the antitumor effects of treatments in relation to chemotherapy [74–76]. Both ERAS and personalized medicine utilize standardized patient information and do not respond to the documented need for person-centred communication [29]. However, questions could be raised about the extent to which person-centeredness could–or should–be contextualized differently in various treatment stages and for different groups of patients. This applies particularly to intervention component I with the interactive PEM, which was designed to apply to address important aspects of treatment and care for patients undergoing CRC surgery, while the second intervention component more generally addresses a transferable person-centred approach to communication. However, considering the principles applied in the development and design process for the novel interactive PEM (see [69]), both components in the intervention are be transferable.

The study design and the development of the person-centred information and communication intervention were informed by suggestions for the development and evaluation of complex clinical interventions [48, 49]. In this way, mapping of conventional care [41–46] was a strength, both to form a praxis-basis for the development of the intervention and for having explicit knowledge about conventional care for the control group. Further, the process oriented evaluations of professionals’ perspective of working with the intervention [52] and how the person-centred communication was applied in consultations [73] are in line with successively obtaining evidence for complex interventions [48], and having knowledge about how an intervention is processed might be of special importance for the development of related implementation strategies [49].

### Limitations

Several limitations should be considered in relation to the design and interpretation of the results. There were changes made in the study protocol as compared to the proposal submitted to the Regional Ethical Review Board half a year before the inclusion of participants started, because the continued analyses of the explorative studies that informed the development the intervention and the design of this evaluation [41–43]. Specifically, the outcome measures were refined. Further, the registration of the study protocol was performed retrospectively and; however, no substantial changes were made to the study design once inclusion of participants had started. The non-randomized design that was based on comparisons before and after the introduction of the intervention contributed to patients being included over an extended period of time (two years and seven months), which may have resulted in other factors influencing the outcomes. Policy decisions included intentions for complicated surgeries to be performed at university hospitals (hospital I) and less complicated in others (hospital II and III). Clearly, the increased use of laparoscopic surgery was identified as a significant difference between the control and intervention group. Changes in clinical practice will always happen and be prioritized before needs for clinical research.

Furthermore, changes in clinical policy and local healthcare organization could have contributed to the reported effect for length of hospital stay. During the study period, it became increasingly more common that patients became hospitalized in the morning at the day for surgery; not the day before surgery. Unfortunately, we have no reliable data about this for all patients, and this is an important consideration in interpreting the result pertaining to length of stay. The increase in laparoscopic surgery for the intervention group could also contribute to the explanation for shorter length of stay; however laparoscopic surgery was controlled for in the analysis.

The functional status domains (except emotional functioning) and global health/quality of life were only based on four time points, and not five like the other self-reported outcome measures. The reason was anticipated special challenges for patients at discharge (time point 2), which made minimizing respondent burden a priority for that time point. However, the results shows that the lowest response rate was actually at time point three. This should be considered in future research involving patients undergoing CRC surgery.

For quasi-experimental designs like the one in this study, a per-protocol analysis is suggested in addition to the intention-to-treat approach. Unfortunately, we found the per-protocol analysis to be impossible due to a lack of reliable information about the extent to which the different intervention components were implemented. Data were obtained about whether patients were exposed to professionals who were knowledgeable about the intervention components, and whether patients had received the PEM, which was part of the intervention. However, there were ambiguities related to the professionals’ application and understanding of the intervention [52], and the qualitative communication analysis based on consultations between patients and nurses indicates variability in communication from *talking with* to *talking to* the patient [73]. This indicates a per-protocol analysis should be based on an assessment of communication in each of the consultations performed with every patient, which would require extensive time and resources.

## Conclusion

This study contributes to the knowledge about challenges for patients undergoing CRC surgery in disclosing the severe impact this treatment has on patients’ recovery process six months following surgery. While the overall hypothesis was not supported, person-centred information and communication intervention was associated with patients’ improved searching for and making use of information, and making sense of the recovery. These effects co-occurred with improved role functioning and change in patients’ behaviours to seek contact with their assigned cancer nurse, instead of contacting a nurse on duty at the ward or visiting an emergency department.

Further, the person-centred information and communication intervention could be regarded as an applicable approach to care that was evaluated in a prevalent group of patients undergoing surgery. The results point to potential benefits of a person-centred care approach to improve information and communication, the discharge process and patients’ recovery following surgery. Further research is suggested on both the contextualization of the novel written interactive PEM in relation to the specific treatment and care processes and the approach for professionals to facilitate person-centred communication in dialogue.

## Supporting information

S1 File TREND statement checklist. (PDF)

S2 File Original project plan for ethical review in June 2012. (PDF)

S3 File Study protocol. (DOCX)
