# Supplementary material for: Preparedness for colorectal cancer surgery and recovery through a person-centred information and communication intervention – A quasi-experimental longitudinal design
Source: PLoS One. 2019 Dec 12;14(12):e0225816. doi: 10.1371/journal.pone.0225816 (PMC6907786; doi:10.1371/journal.pone.0225816)
Supplement: S2 File — (PDF) [file pone.0225816.s002.pdf]

2012 -06- 2 5

Bilaga 2

536-12

Tailored Information Materials and Person-centred Communication – An Intervention for Improved Recovery Following Colorectal Cancer Surgery

SPECIFIC GOALS

The aim is to investigate whether tailored information material and person-centred communication for patients diagnosed with colorectal cancer (CRC) undergoing elective surgery can improve the patients' recovery following diagnosis and initial treatment.

The specific goals are as follows.

- To evaluate whether the intervention with tailored information material and person-centred communication intervention will enhance health-related quality of life (HRQoL), communication and attitudinal self-efficacy and well-being during recovery
- To explain variations in patients' recovery trajectories during the six months following CRC surgery through intersections of diverse socioeconomic, disease-, health- and illness-related aspects and aspects related to the cancer care process, and from such variations identify particularly vulnerable sub-groups of patients

BACKGROUND

Each year, approximately 3,900 people are diagnosed with colon cancer and 1,900 with rectal cancer, split almost equally between women and men. The primary treatment for colorectal cancer (CRC) is surgery. In CRC surgery, 'enhanced recovery' is a major influencing concept, designed to minimise the stress response associated with pre-, peri- and postoperative treatments[1]. In CRC, it is implemented nationally in an increasing number of institutions using a multimodal approach, including special pre-surgery information, nutrition, pain management and active mobilisation leading to a reduced hospital stay[1]. Enhanced recovery after surgery (ERAS) has been positively evaluated in CRC care.[2] However, ERAS focuses on somatic aspects and standardised patient information[2] and research on patient-reported outcomes reveals a lack of patient participation in care and that support from professionals is crucial.[3] In addition, ERAS does not respond to the socioeconomic differences among patients with CRC[4].

A particular challenge in CRC care is meeting patients' information needs over time in order to improve recovery. However, the focus in cancer communication research has been patient–provider communication in dyads.[5] With such an approach, outcomes in cancer care are modest or low[6], despite considerable efforts to promote knowledge transfer.[7] There is thus a need for comprehensive considerations and complex interventions over time.[8]

Recovery after CRC surgery. For patients diagnosed with gastrointestinal cancer, "recovery" in the form of regaining control over biopsychosocial functions [9] is related to changes over time in both coping strategies and emotional well-being.[10, 11] This implies that patients undergo a variety of recovery trajectories but how these are shaped has not yet been described. Information about such trajectories might form a basis for tailoring interventions to meet patients' information needs related to CRC surgery.

Recovery after surgical procedures has been studied mainly by measuring clinical parameters, e.g. length of stay, symptoms. During the last decade, the length of hospital stay has been reduced considerably, especially with the ERAS protocol. As a result, much of the responsibility for postoperative recovery is being shifted to the patients and their families and a large part of the recovery is taking place in the patient's home without help and support from hospital staff. This imposes considerable demands on patients to manage their daily lives and presupposes that patients are well informed.[3, 12]

ERAS gives patients no time to reflect on what recovery at home following discharge entails. Furthermore, for the majority of patients, emotional reactions related to postoperative recovery are reported during a later phase when their physical condition was under control.[12] It should be noted that the ERAS protocol does not include what happens during the later phase of recovery at home. When diagnosed with cancer, fear of death is precipitated and the anxiety of being constantly confronted with this threat of death is aroused[13] and the major emotional reactions appear after discharge from hospital. The emotional suffering represents an existential aspect of the postoperative recovery process[12] and patients with CRC have also reported existential uncertainty before surgery.[14] However, successful recovery is found to require information that is in line with the patient's individual needs and health literacy.[15]

Tailored information and person-centred communication. The project draws on the results of cancer communication from the Swedish State of the Science Conference in 2009; patient–provider communication as interaction in a communicative environment. [5] This will be put in relation to (a) the patient's need for suitable and comprehensible written information[35] as a supplement to verbal information,[16] (b) the patient's need for information along the cancer journey[8] and routines in person-centred care.[17] In this way, the extant knowledge in cancer care communication will be built on.

A meta-analysis presents limited evidence of interventions delivered before consultations to help patients address their information needs in consultations.[18] Training clinicians in addition to patient interventions presents no clear benefits. In 26 of the 33 studies, the intervention was delivered to the patients immediately before the consultation in the waiting room, which the authors consider to represent mechanistic "quick fixes". The inclusion of nurse–patient communication in addition to physician–patient communication was identified as the only exception.[18] A recent pilot study demonstrated the effectiveness of nurse-delivered telephone supportive intervention with improvements in total quality-of-life change scores six months after surgery.[19] For this reason, more comprehensive interventions that address patient complexities in patient information and communication are suggested,[18] viewing information as a continuous process[16] and including specialist nurses providing and commenting on information to the patient.[18]

Person-centred care aims to advance concordance between care provider and patient on the treatment plan by using the patient narrative as the starting point, and to focus on the patient's point of view and sense-making, establish shared decision-making and secure documentation of patient's preferences, beliefs and values.[17]

Patient recovery outcomes. In order to depict the factors influencing recovery, we have chosen the revision by Ferrans et al[20] of the HRQOL conceptual model by Wilson and Cleary,[21] suggesting five types of measures for patient outcomes. Here these measures will be adapted to the situation for patients with CRC: (1) biological function; CRC and treatment-oriented variables, (2) symptoms; patients' experience, (3) functional status; coping efforts, self-efficacy, emotional function, and preparedness for surgery and recovery (4) general health perceptions; HRQoL, and (5) overall quality of life; wellbeing. The model suggests the earlier types of measures to influence the subsequent measures. Furthermore, all five types of measures are considered to be influenced by the characteristics of the individual and the environment. Here, the characteristics of the individual include gender, age and coping capacity. Characteristics of the environment include CRC care process variables and socio-economic factors.

WORKING PLAN AND PRELIMINARY RESULTS

Hypotheses

The hypotheses are:

1. meeting patients' information needs and preparing patients for surgery and recovery through tailored information material and person-centred communication will lead to improved HRQoL and well-being, as well as communication and attitudinal self-efficacy (primary outcomes) and improved emotional function, preparedness for surgery and recovery and decreased symptom experience (secondary outcomes)
2. variations in patients' recovery trajectories during the six months following CRC surgery could be explained by intersections of diverse socioeconomic, disease-, health- and illness-related aspects and that such variations in recovery can be used to identify particularly vulnerable sub-groups of patients

Considerations for the design

For cancer care information and communication research, complex interventions with various interconnecting components are suggested.[5, 8] A sequential approach for designing and evaluating complex interventions is suggested based on evidence from combinations of qualitative and quantitative data,[22, 23] thus, multiphase mixed methods designs with fully integration of qualitative and quantitative methods across the research process.[24]

Study design

Figure 1 provides an overview of the study design. Ongoing pre-studies (see below, preliminary results) are used for planning the intervention. The intervention will be developed with the active participation of CRC patients and CRC teams. Two systems for information and communication will be compared – standard care versus tailored information materials and person-centred communication – in a quasi-experimental longitudinal design. Briefly, an initial 280 patients at three hospitals will be included in standard care: the control group.

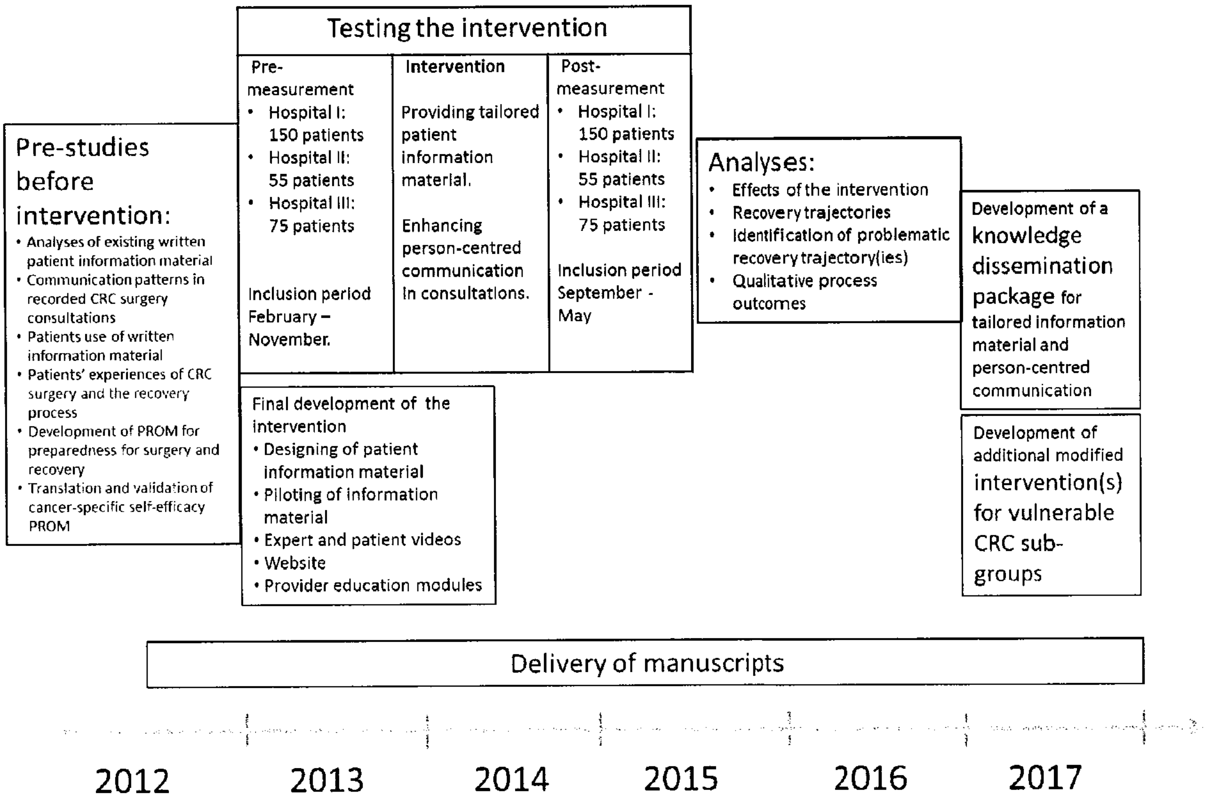

Figure 1. Overview of the design.

The tailored information material and person-centred communication will then be implemented and evaluated by means of inclusion of a further 280 patients; the intervention group. The impact of the intervention will be evaluated by patient-reported outcomes measures (PROM) across five time points pre- and post-surgery (see table p. 7). The results of the intervention will be used for (a) development of a knowledge dissemination package for tailored information material and person-centred communication, and (b) for the development of additional modified intervention(s) for vulnerable CRC sub-groups.

Preliminary results
Ongoing analyses from explorative pre-studies, started in the winter of 2011, reveal problematic aspects of standard CRC care:

- *Levelling of written patient information material: unsuitable and adequate, but superior only in exceptional cases. No materials present the CRC care process in relation to patients' daily lives.* Existing written materials from 28 Swedish surgical departments are included and analysed using language technology methods and the validated instrument SAM+CAM,[36] These preliminary results will be complemented with patients' opinions on the matter from focus groups.
- *Written information material pertaining to enterostoma: tend to clearly include descriptions of informational as well as participatory genres, while all other materials tend to include only informational genres.* Discourse analysis of the same material as above. Participatory genres may be particularly significant for describing patients' CRC journey over time.
- *Patients' experience: problems managing the recovery process on a personal level and would like pribnted and online material, including explanation of the CRC care process supported by verbal information, preferably from nurses. Even well-educated patients would like easily read materials at the times they are emotionally involved.* Data from five focus groups with patients and narrative interviews with seven patients (strategic sampling) after recovery from CRC surgery. The preliminary result implies health literacy or preferences regarding the level of information material cannot be assessed at a pre-defined time. Some patients did not consider themselves to have recovered from surgery until six months later.
- *Patient–provider consultations before and after CRC surgery: reveal a clear agenda, although not explicated for patients. In what way patients make sense of the CRC and recovery from surgery is only included vaguely. In consultations, professionals tend to act as if they are taking part in patient's single events rather than in a series of sequential activities, and thus not taking full responsibility for actively transferring patients to the next provider in line. This is most evident in the discharge from hospital after surgery.* Prospective interaction data from consultations at four time points with seven patients before and after CRC surgery.

In addition to these ongoing analyses, the qualitative interview and interaction data have been used for selection and development of instruments for PROM (further described below).

Intervention
The intervention aims to enhance a movement from patients receiving information and instructions passively to being active participants in their recovery process. Special attention will be given to focusing on preparing patients for surgery and recovery over time and for participation in recovery. The development of the intervention will be based on the results from the ongoing pre-studies (patients' experiences), previous international research evidence and CRC surgery care experts. The development of tailored information material will build on the research tradition in Language Technology and Linguistics on readability,

comprehensibility, and health literacy, and research on tools that aid the authoring process[25] and natural language generation.[26] The intervention consists of two interrelated components:

1. *Providing tailored patient information material.*
   - New written information material will be designed based on a toolkit from the pre-studies of written materials (see above). In existing written patient information material topics will be supplemented with the CRC care process related to patients' daily lives and will include examples of patients' experiences over time.
   - Access to a web-based computational tool that will support the creation of *adaptive information material.* Based on the idea of a*daptive information material* (an abstract representation of the *available* information material) a set of parameters may be rendered into different tailored texts, varying in, e.g., information density or terminology; easy reading as well as detailed and comprehensive versions. The tool will also support the authoring process by automatic linguistic analysis of the target text where it will highlight potential problems, such as the overuse of passive constructions or complex sentence structure.
   - The information material will be provided as a printed version and on a website, with options for sequential access (pre-surgery, at discharge, during recovery).
   - Videos with interviews with experts and former patients as well as links to valid resources on the web will be included on the website, i.e. sorted and packed valid information will be provided.

2. *Enhancing person-centred communication in consultations.*
   - Developed "contact nurse" function. The contact nurses in particular will use the patient narrative as the starting point[17] for guidance of patients' sense-making of preparedness for surgery, recovery on discharge, (with a telephone call) at home following discharge, and at the return visit (at the surgical clinic). This will be facilitated by:
     1. Providing a special two-day training programme for the contact nurses.
     2. Clinical supervision to the contact nurses. Monthly meetings for two hours in groups (one at each of the three hospitals), with a specialist nurse trained in clinical supervision, during the intervention period. The focus of the supervision will be on how to work with the patient narrative in person-centred care, in consultations and in documentation in patient records.[17]
   - CRC team professionals actively using and referring to patient information material. Two workshops will be held and videos developed for CRC teams and staff caring for patients during surgery, dealing with how the new information material is designed and provided. Examples of how it can be used to enable patients to play an active part in their recovery process will be given and discussed.

Sampling and data collection

A total of 560 patients eligible for CRC elective surgery will be included. Exclusion criteria: receiving long-term radiotherapy or chemotherapy initially after CRC diagnosis. Due to the exploratory nature of the trajectory analyses, it is not feasible to determine a priori the number of variables and the magnitude of the relationships among them that would be needed to determine the sample size requirements for the multivariate statistical models. However, based on the general rule of thumb of requiring a minimum of 10 to 20 subjects per variable[27], it is anticipated that the sample size will be adequate for the simultaneous analysis of about 22-45 observed variables in the combined sample. Once specific analytical models have been determined, power analyses will be conducted using Monte Carlo simulation to confirm sample size requirements with regard to the specified statistical models.[28] Standard care (first group) 280 patients, followed by implementation of the intervention model (second group)

280 patients. To enable a longitudinal analysis, questionnaires will be sent out to patients five times: (1) before surgery (at pre-surgery information), (2) at discharge after surgery, (3) six weeks after surgery, (4) three months after surgery and (5) six months after surgery.

A study-specific case report formula will be developed for demographic, disease and treatment-related data to be collected from the patient records. Validated reliable instruments have been selected out of data from the ongoing pre-studies (above, preliminary results). Included is a cancer-specific, validated instrument for self-efficacy[29], which we have translated into Swedish. Further, based on the data, an instrument for "patient preparedness for CRC surgery and recovery" is has been developed and adapted to five time points with 25-34 items. Both instruments are currently undergoing expert panel evaluations and validations.[37]

The following instruments will be used to measure variables corresponding to each of the concepts in the modified Wilson and Cleary model:

**Concept**	**Variable**	**Instrument**	**Before surgery**	**Before discharge**	**6 weeks after**	**3months after**	**6 months after**
Biological function	CRC diagnosis, type of surgery, adjuvant therapy	From patient records	X	X	X	X	X
Symptoms	Symptom experience	EORTC QLQ-C30[30]	X		X	X	X
Functional status	Coping efforts<br>Self-efficacy<br>Preparedness for surgery and recovery	Daily coping assessment[31]<br>CASE[29]<br>Under development	X<br>X<br>X		X<br>X<br>X	X<br>X<br>X	X<br>X<br>X
General health perceptions	HRQoL	EORTC QLQ-C30[30]	X		X	X	X
Overall QoL	Well-being,	EORTC QLQ-C30[30]					
Individual character.	Coping capacity<br>Gender, age, social backgrou.	SoC – 13[32]<br>Single items	X<br>X				X
Environm. character.	Social living conditions & employment sit., CRC care process	Single items & patient records	X				

To evaluate process outcomes, narrative interviews and recording of consultations with seven patients in the intervention group will be conducted – the same as the principles used in the pre-studies. Sample comparison regarding clinical variables to data from the national population of patients from the CRC National Quality Registries will be performed.

Processing and analysis

Confirmatory factor analysis will be used to assess construct validity and reliability of PROM that involve multiple items. Descriptive statistics will be used to evaluate PROM and for correlations with socioeconomic, disease and illness-oriented variables. The outcome

variables will be represented as repeated measures to evaluate the change from baseline to six weeks and three and six months post-surgery. Change scores in outcome variables, will be compared between the intervention and control groups while controlling the potentially confounding effects of other extraneous variables. A repeated-measures ANOVA procedure will be employed to evaluate the differences in the outcome variables between groups across the five time points (hypothesis 1). We will check specifically for clinical differences, including: (a) whether patients continue with adjuvant chemotherapy or any other additional treatments, and (b) CRC clinical factors, including diagnosis and type of surgery.

Multi-level (mixed effects) longitudinal models will be specified to compare the trajectories of the relevant outcomes between the intervention and control groups (hypothesis 2). Selection of the outcome variables for the analyses of trajectories will be based on evaluation of the relative magnitudes of the change scores. Trajectories from the time of pre-surgery information and the following three and six months will be differentiated[33, 34] and then interpreted qualitatively by applying qualitative narrative, case-oriented data from narrative interviews and consultations with patients in standard groups and intervention groups.[24]

Sub-group(s) of patients presenting problematic recovery trajectories will be identified from the trajectory analysis. In addition, process evaluation of the intervention will be performed through qualitative, comparative analysis of narrative interviews and recorded consultations. Analyses similar to those in the ongoing pre-studies will be used for this purpose.

Development of a knowledge dissemination package for tailored information material and person-centred communication

The tested intervention will be modified from the results from the study. The components and strategies utilised in the intervention will be developed into a package to be used for clinical implementation of the intervention. Here, the recovery trajectories and the qualitative process evaluation will be of special significance in order to enable sustainable clinical development. The research team's established collaboration with the Regional Cancer Centre West will be primary partners in this dissemination development in order to reach a clinically useful package. The web based tool will be integrated into Språkbanken's sustainable research infrastructure and made publicly available (spraakbanken.gu.se).

Development of additional modified intervention(s) for vulnerable CRC sub-groups

The problematic recovery trajectories may reveal vulnerable sub-group(s) of patients undergoing CRC surgery. If the results indicate this, the data and results will be sufficient to demonstrate in what respect the tested intervention was less efficient for such group(s). Specific modifications for the group(s) will be developed. Specific modified intervention(s) will be designed for further research evaluation.

International collaboration

Included in the research team is Associate Professor Rick Sawatzky from Trinity Western University in Vancouver, Canada. He will contribute with expertise in the analysis of PRO- and quality of life outcomes.

The project will be conducted at two research centres: (a) the University of Gothenburg Centre for Person Centred Care, which means a valuable environment for inter-disciplinary healthcare research with rich potential for additional local, regional, national and international collaboration with highly recognised scholars, and (b) the Palliative Research Centre at Ersta Sköndal University and Ersta Hospital in Stockholm, which is a newly established centre with special potential for clinical research.

The researchers in the team have several national and international collaborative partners. Professor Srikant Sarangi from Cardiff, UK (communication studies), collaborates with

Kokkinakkis, Hydén and Rystedt and will meet Eva Carlsson in June 2012. Professor Sally Thorne, an expert in cancer care communication from the School of Nursing, University of British Columbia, Vancouver, Canada, has ongoing contact with Öhlén and Friberg. Friberg was a co-organiser and president of the first conference in Sweden "State of the science in patient education and learning", in January 2011, and has established collaboration with two of the keynote speakers: Professor Barbara Klug Redman, Wayne University, Detroit, Michigan, USA, and Professor Helena Leino Kilpi, University of Turkku, Finland. Team members participate actively in research networks, including Swedish Researchers in Cancer Care, which involves additional international collaborative partners.

SIGNIFICANCE

Tailored information material and person-centred communication in cancer care will be particularly significant in the media society, as an increase can be expected in the importance of enabling patient participation in healthcare. The model will be adapted to support patients' knowledge-seeking and knowledge management even in information- and knowledge-compact contexts.

The intervention will be targeted for patients diagnosed with CRC who are undergoing elective surgery. For CRC treatments, the model will be significant in relation to the somatic focused concept of ERAS. Patients undergoing CRC surgery are a diverse group of patients and the discrimination of patients' recovery trajectories based on intersections of somatic and psycho-socio-economic variables will provide a significant basis for further development of additional interventions for specific sub-groups of patients with CRC. Although CRC is a common cancer diagnosis, affecting both women and men, the care related to CRC is comparatively under-researched. The proposed research will therefore contribute to evidence-based care.

The intervention model will help improving the quality and consistency of an information material by automatic linguistic and statistical analysis, and it will enable the creation of an adaptive information material with the added benefit that will ultimately increase a patient's capacity to self-manage and most importantly patient outcomes and safety. Further, a model based on explanatory knowledge will be significant to practical and sustainable implementation of the model.

There are national medical guidelines for CRC and only two regional CRC nursing programmes. The programme contains an overall structure but lacks strategies for information and communication, since there is a lack of evidence for it. These existing guideline and care programmes, together with the knowledge dissemination package that is to be delivered, will facilitate implementation.

Today, the majority of people diagnosed with CRC can expect five-year survival. This implies that healthcare needs to meet patients' concerns about different treatment options available in new ways compared with previously. The intervention with tailored information and person-centred communication is likely to be easily adapted and applied beyond elective surgery.

Together with the researchers' studies dealing with information and communication related to advanced gastrointestinal cancer, the results will constitute a significant basis for the development of an information and communication model for colorectal cancer encompassing, and adapted to, the different phases – from diagnosis to rehabilitation and palliation.

REFERENCES

1. Kehlet H. Multimodal approach to control postoperative pathophysiology and rehabilitation. Br J Anaesth. 1997;78(5):606-617.
2. Eskicioglu C, Forbes SS, Aarts MA, Okrainec A, McLeod RS. Enhanced recovery after surgery (ERAS) programs for patients having colorectal surgery: a meta-analysis of randomized trials. J Gastrointest Surg. 2009;13(12):2321-2329.
3. Norlyk A, Harder I. After colonic surgery: The lived experience of participating in a fast-track programme. International journal of qualitative studies on health and well-being. 2009;4:170-180.
4. Cavalli-Bjorkman N, Lambe M, Eaker S, et al. Differences according to educational level in the management and survival of colorectal cancer in Sweden. Eur J Cancer 2011;47(9):1398-406.
5. Brundage MD, Feldman-Stewart D, Tishelman C. How do interventions designed to improve provider-patient communication work? Illustrative applications of a framework for communication. Acta Oncol. 2010;49(2):136-143.
6. Butler L, Degner L, Baile W, Landry M. Developing communication competency in the context of cancer: a critical interpretive analysis of provider training programs. Psychooncology. 2005;14(10):861-872; discussion 873-864.
7. Thorne SE, Bultz BD, Baile WF. Is there a cost to poor communication in cancer care?: a critical review of the literature. Psychooncology. 2005;14(10):875-884; discussion 885-876.
8. Street AF, Horey D. The State of the Science: Informing choices across the cancer journey with public health mechanisms and decision processes. Acta Oncologica. 2010;49(2):144-152.
9. Allvin R, Berg K, Idvall E, Nilsson U. Postoperative recovery: a concept analysis. J Adv Nurs. 2007;57(5):552-558.
10. Nordin K, Glimelius B. Reactions to gastrointestinal cancer--variation in mental adjustment and emotional well-being over time in patients with different prognoses. Psychooncology. 1998;7(5):413-423.
11. Winterling J, Wasteson E, Glimelius B, Sjoden PO, Nordin K. Substantial changes in life: perceptions in patients with newly diagnosed advanced cancer and their spouses. Cancer Nurs. 2004;27(5):381-388.
12. Allvin R, Ehnfors M, Rawal N, Idvall E. Experiences of the postoperative recovery process: an interview study. Open Nurs J. 2008;2:1-7.
13. Northouse LL, Schafer JA, Tipton J, Metivier L. The concerns of patients and spouses after the diagnosis of colon cancer: a qualitative analysis. J Wound Ostomy Continence Nurs.1999;26(1):8-17.
14. Moene M, Bergbom I, Skott C. Patients' existential situation prior to colorectal surgery. J Adv Nurs. 2006;54(2):199-207.
15. Allvin R. *Postoperative Recovery: Development of a Multi-dimensional Questionnaire for Assessment of Recovery*. Örebro: Hälsoakademin, Örebro universitet; 2009.
16. Beaver K, Campbell M, Craven O, Jones D, Luker KA, Susnerwala SS. Colorectal cancer patients' attitudes towards involvement in decision making. Health expectations. 2009;12(1):27-37.
17. Ekman I, Swedberg K, Taft C, et al. Person-centered care--ready for prime time. European journal of cardiovascular nursing : journal of the Working Group on Cardiovascular Nursing of the European Society of Cardiology. 2011;10(4):248-251.
18. Kinnersley P, Edwards A, Hood K, et al. Interventions before consultations for helping patients address their information needs. Cochrane Database Syst Rev. 2007;18(3):CD004565.

19. Harrison JD, Young JM, Solomon MJ, Butow PN, Secomb R, Masya L. Randomized pilot evaluation of the supportive care intervention "CONNECT" for people following surgery for colorectal cancer. Diseases of the colon and rectum. 2011;54(5):622-631.
20. Ferrans CE, Zerwic JJ, Wilbur JE, Larson JL. Conceptual model of health-related quality of life. J Nurs Scholarsh. 2005;37(4):336-342.
21. Wilson IB, Cleary PD. Linking clinical variables with health-related quality of life. A conceptual model of patient outcomes. Jama.1995;273(1):59-65.
22. Campbell M, Fitzpatrick R, Haines A, et al. Framework for design and evaluation of complex interventions to improve health. Bmj.16 2000;321(7262):694-696.
23. Craig P, Dieppe P, Macintyre S, Michie S, Nazareth I, Petticrew M. Developing and evaluating complex interventions: the new Medical Research Council guidance. Bmj. 2008;337:a1655.
24. Morse JM. Simultaneous and sequential qualitative mixed method designs. Qual Inquiry. 2010;16(6):483-491.
25. Oelke D, Spretke D, Stoffel A, Keim DA. Visual Readability Analysis: How to make your writings easier to read. Paper presented at: IEEE Conference on Visual Analytics Science and Technology, 2010.
26. Hunter J, Freer Y, Gatt A, et al. BT-Nurse: Computer Generation of Natural Language Shift Summaries from Complex Heterogeneous Medical Data. J American Med Info Ass. 2011;18:621-624.
27. Schumacker RE, Lomax RG. A beginner's guide to structural equation modeling. 3rd ed ed. New York: Routledge; 2010.
28. Enders C. Analyzing structural equation models with missing data. In: Hancock G, Mueller R, eds. Structural Equation Modeling: A Second Course. Greenwich: Information Age Publishing; 2006:313-342.
29. Wolf MS, Chang CH, Davis T, Makoul G. Development and validation of the Communication and Attitudinal Self-Efficacy scale for cancer (CASE-cancer). Patient education and counseling. 2005;57(3):333-341.
30. Uwer L, Rotonda C, Guillemin F, et al. Responsiveness of EORTC QLQ-C30, QLQ-CR38 and FACT-C quality of life questionnaires in patients with colorectal cancer. Health and quality of life outcomes. 2011;9:70.
31. Wasteson E, Glimelius B, Sjödén P, Nordin K. Comparison of a questionnaire commonly used for measuring coping with a daily-basis prospective coping measure. J Psychosom Res. 2006;61(6):813-820.
32. Eriksson M, Lindstrom B. Antonovsky's sense of coherence scale and its relation with quality of life: a systematic review. Journal of epidemiology and community health. 2007;61(11):938-944.
33. Heck RH, Thomas SL. An introduction to multilevel modeling techniques. Vol 2nd Ed. New York: Routledge; 2009.
34. Shin JH. Application of repeated-measures analysis of variance and hierarchical linear model in nursing research. Nursing research. 2009;58(3):211-217.
35. Finnie RK, et al. Beyond reading level: a systematic review of the suitability of cancer education print and Web-based materials. J Cancer Educ 2010;25(4):497-505.
36. Helitzer D, et al. Health literacy demands of written health information materials: an assessment of cervical cancer prevention materials. Cancer Control. 2009;16(1):70-8.
37. Wild D, et al. Principles of Good Practice fot the Translation and Cultural Adaption Process for Patient-Reported Outcomes (PRO) Meaures: Report of the ISPOR Task Force for Translation and Cultural Adaption. Value in health 2005; 8(2), 94-104.
