# Supplementary material for: Preparedness for colorectal cancer surgery and recovery through a person-centred information and communication intervention – A quasi-experimental longitudinal design
Source: PLoS One. 2019 Dec 12;14(12):e0225816. doi: 10.1371/journal.pone.0225816 (PMC6907786; doi:10.1371/journal.pone.0225816)
Supplement: S3 File — (DOCX) [file pone.0225816.s003.docx]

**Intervention group**

March 2014 to June 2015

**Control group**

November 2012 to January 2014


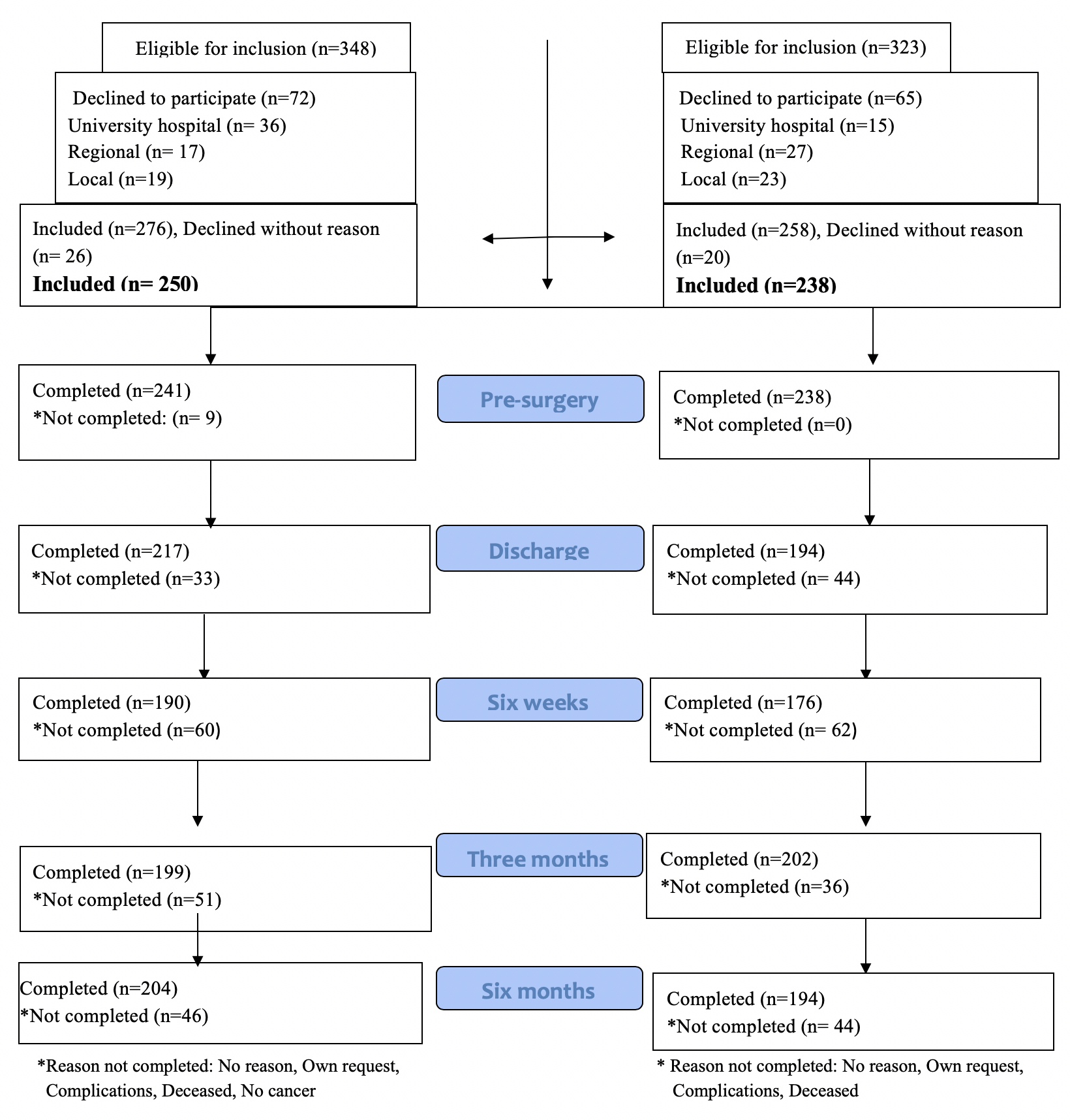


Fig 1. Study Flow chart.


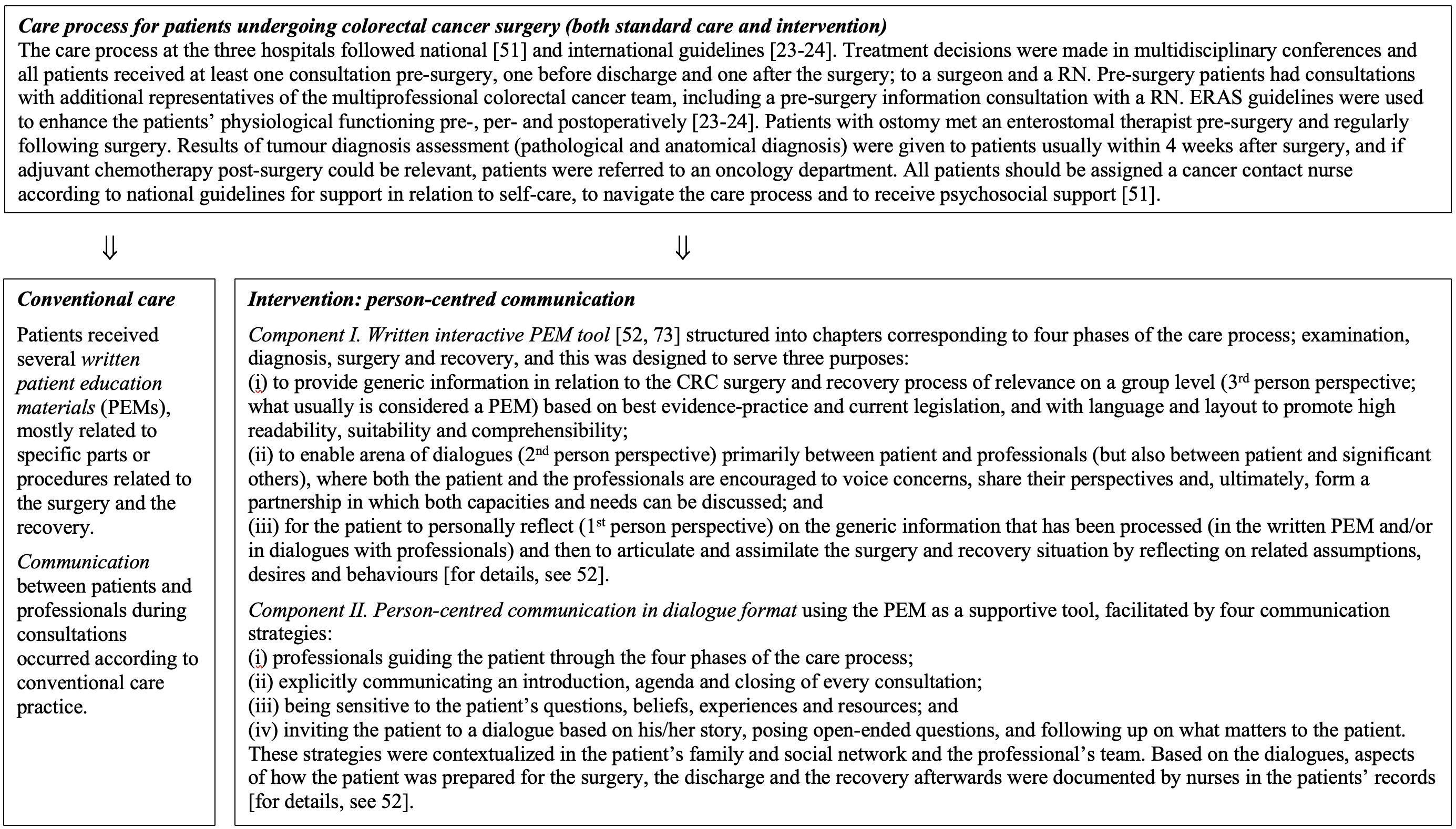


Fig 2. Conventional care and intervention components as related to the common care process.

Fig 3. Specific intervention events as related to selected events in the care process for patients undergoing CRC surgery, and data collection time points.

*Note*. ^*^ Part 1 of the PEM (chapter 1) was delivered to the patients in the intervention group directly after inclusion, and Part 2 (chapters 2-4) was delivered when the patient had received the cancer diagnosis. For chapter topics see Fig 2. For additional details of ways the intervention PEM was to be used in the intervention see [52, p. 4].

Fig 4. Trajectories of patient preparedness for surgery and recovery (N = 488)

|  |  |
| --- | --- |
|  |  |
|  | |
| *Notes.* Time points: 1 = Pre-surgery, 2 = Discharge, 3 = 4-6 weeks after surgery, 4 = 3 months after surgery, 5 = 6 months after surgery. | |

Fig 5. Trajectories of global health/quality of life, cognitive, emotional, role, social, and physical functioning (N = 488).

|  |  |
| --- | --- |
|  |  |
|  |  |
|  | |

*Notes.* Time points: 1 = Pre-surgery, 2 = Discharge, 3 = 4-6 weeks after surgery, 4 = 3 months after surgery, 5 = 6 months after surgery. Only the Emotional functioning subscale of the EORTC was administered at time point 2 (discharge).

Fig 6. Trajectories of distress (N = 488)

*Notes.* Time points: 1 = Pre-surgery, 2 = Discharge, 3 = 4-6 weeks after surgery, 4 = 3 months after surgery, 5 = 6 months after surgery.
